# LAPTM4B-mediated hepatocellular carcinoma stem cell proliferation and MDSC migration: implications for HCC progression and sensitivity to PD-L1 monoclonal antibody therapy

**DOI:** 10.1038/s41419-024-06542-8

**Published:** 2024-02-22

**Authors:** Haojun Wang, Quanwei Zhou, Ding Fang Xie, Qingguo Xu, Tongwang Yang, Wei Wang

**Affiliations:** 1 Department of Urology, Beijing Chaoyang Hospital, Capital Medical University, 100020 Beijing, China; 2 Capital Medical University, 100071 Beijing, China; 3 The National Key Clinical Specialty, Department of Neurosurgery, Zhujiang Hospital, Southern Medical University, Guangzhou, China; 4 The Second Department of Medical Oncology, Xiangtan Central Hospital, Xiangtan, China; 5 Department of Organ Transplant Center, The Affiliated Hospital of Qingdao University, Qingdao, China; 6 The Hunan Provincial University Key Laboratory of the Fundamentaland Clinical Research on Functional Nucleic Acid, Changsha Medical University, Changsha, China

**Keywords:** Cancer, Stem cells, Cancer

## Abstract

In hepatocellular carcinoma (HCC), immunotherapy is vital for advanced-stage patients. However, diverse individual responses and tumor heterogeneity have resulted in heterogenous treatment outcomes. Our mechanistic investigations identified LAPTM4B as a crucial gene regulated by ETV1 (a transcription factor), especially in liver cancer stem cells (LCSCs). The influence of LAPTM4B on LCSCs is mediated via the Wnt1/c-Myc/β-catenin pathway. CXCL8 secretion by LAPTM4B drove myeloid-derived suppressor cell (MDSC) migration, inducing unfavorable patient prognosis. LAPTM4B affected PD-L1 receptor expression in tumor microenvironment and enhanced tumor suppression induced by PD-L1 monoclonal antibodies in HCC patients. LAPTM4B up-regulation is correlated with adverse outcomes in HCC patients, sensitizing them to PD-L1 monoclonal antibody therapy.

## Introduction

Hepatocellular carcinoma (HCC) is the third leading cause of cancer-related death worldwide [1], with a 5-year survival rate of 18% [2]. For early HCC patients, treatments include liver resection, radiofrequency ablation, and liver transplantation. However, advanced-stage patients are mainly treated with systemic treatments. Immunotherapy, particularly using immune checkpoint inhibitors (ICIs) like programmed cell death protein 1 (PD-1) and programmed cell death ligand 1 (PD-L1) monoclonal antibodies, represents a significant breakthrough in treating solid tumors, including HCC [3]. However, the objective response rate of these drugs as monotherapies for HCC is only 15-20% [4], and other patients experience rapid tumor progression. Although immune characteristics predicting tumor responses to ICIs have been identified, reliable biomarkers are lacking. Therefore, it is urgent to provide new theoretical basis for anti-liver cancer immunotherapy through biomarkers.

Lysosomal-associated transmembrane protein 4B (LAPTM4B), located on chromosome 8q22.1, encodes a membrane protein with four transmembrane regions [5, 6]. It is a binding site for SH3 domain of particular signaling molecules and is important for tumor cell proliferation and metastasis [7, 8]. LAPTM4B is explored in ischemia-reperfusion injury, but studies mainly focus on its role in cancer [5]. LAPTM4B is overexpressed in solid tumors, including breast cancer, non-small-cell lung cancer, ovarian cancer, gastric cancer and HCC, and predicts poor prognosis [9–13]. However, the crucial mechanisms associating LAPTM4B expression with HCC occurrence and progression, and its relationship with immunotherapy, remain unclear.

Myeloid-Derived Suppressor Cells (MDSCs) are distinct immune cells, whose numbers are significantly increased in pathologies including cancer, chronic infections, and inflammation. MDSCs are crucial for immune suppression and tolerance, inhibiting T cell activity, and aiding immune escape of tumor cells. After accumulation and activation in bone marrow, MDSCs are attracted to tumors through chemokine-chemokine receptor interactions in tumor microenvironment (TME) [14]. MDSC occurrence and development are associated with HCC [15]. Nevertheless, the mechanism of LAPTM4B in inducing MDSCs is unclear.

This study uncovers the upstream promoter ETV1 and its binding sequence for LAPTM4B. LAPTM4B overexpression promotes liver cancer stem cell (LCSC) proliferation and MDSC migration. This approach is beneficial for the conjunction with PD-L1 antibodies.

## Materials and methods

### Patients and animals

From March 2014 to August 2020, 92 pairs of tumor and adjacent tissue samples were collected from HCC patients at The Affiliated Hospital of Qingdao University; An additional 187 pairs of samples were obtained from Eastern Hepatobiliary Surgery Hospital Affiliated to Naval Medical University. Paraffin sections from 21 HCC patients receiving PD-L1 mAb at The Affiliated Hospital of Qingdao University between May 2017 and August 2020 were included. Peripheral blood mononuclear cells (PBMCs) were sourced from healthy donors. Participants provided written consents, and the study adhered to Helsinki Declaration guidelines. Approval had been obtained from the Clinical Research Ethics Committee of The Affiliated Hospital of Qingdao University and Eastern Hepatobiliary Surgery Hospital Affiliated to Naval Medical University.

Six-week-old male *BALB/c* mice (SPF (Beijing) Biotechnology Co., Ltd.) were housed in an SPF animal facility. Animal experiments were approved by the Ethics Committee of Changsha Medical University (Approval No: 2023038) and conducted following the Animal Center of Changsha Medical University and National Institutes of Health guidelines.

### HCC mouse model

At one week of age, mice were subjected to intraperitoneal injections of DEN, administered every 7 days for 4 injections. The mice were raised until they reached ten months of age. The mice were euthanized humanely, and tissue samples were collected for further analysis.

### Hematoxylin and Eosin staining

After formalin fixation, tissues were dehydrated with alcohol, infiltrated with paraffin wax, sliced into thin sections, mounted onto glass slides, de-waxed for paraffin removal, stained with hematoxylin (cell nuclei were stained blue-purple), and eosin (cytoplasm was stained pink), dehydrated and transparentized. A cover slip was applied using a transparent adhesive, and stained tissue sections were preserved.

### Immunohistochemistry

The tissue slides were initially baked at 60 °C for 12 h. The following steps were executed: deparaffinization using xylene and ethanol, antigen retrieval in citrate buffer, and blocking endogenous peroxidase activity with periodic acid. Primary antibody (LAPTM4B) was applied overnight, succeeded by incubation with anti-rabbit IgG antibody-HRP polymer. DAB staining was performed, and counterstaining was done using hematoxylin. The sections underwent dehydration, xylene immersion, and were mounted with neutral resin for microscopic examination.

### Multiplex immunofluorescence

Tissue sections were deparaffinized using xylene and ethanol, followed by antigen retrieval with EDTA buffer and microwave heating. Endogenous peroxidase activity was blocked, and nonspecific binding was prevented with BSA. After primary antibody incubation, sections were incubated with HRP-conjugated secondary antibody. Detection was performed using CY3-TSA and FITC-TSA. Nuclei were stained with DAPI and examined microscopically with imaging.

### Electrophoretic Mobility Shift Assay

The biotin-labeled Electrophoretic Mobility Shift Assay (EMSA) probe was obtained. A 4% EMSA gel was prepared. In the EMSA binding reaction, the probe and proteins were incubated, and the mixture was loaded into wells. Electrophoresis was conducted using 0.5XTBE buffer. The gel and transferred nylon membrane were UV-crosslinked. The membrane was blocked, treated with Streptavidin-HRP Conjugate, washed, and exposed to BeyoECL Moon working solution. The chemiluminescent signals were detected using a Western blot imager.

### Cell culture

HepG2, Huh7, and Hep3B cells were obtained from Chinese Typical Culture Collection Center, confirmed by STR analysis and grown in DMEM or MEM with 10% fetal bovine serum and 1% streptomycin/penicillin.

### PBMC separation

Blood was sampled into 15-ml tubes and centrifuged at 2000rpm. Then, 1x PBS was mixed with the resultant diluted cell pellet. After introducing lymphocyte separation medium into PBMC separation tube, whole blood was layered. After being centrifugated at 2000rpm for 10 min, we meticulously collected and washed cells twice with 1x PBS. Red blood cells were eliminated with red blood cell lysis buffer, and washed with 1x PBS twice for purity.

### Vector construction

To prepare the lentivirus, a Lentiviral transfer vector (pLV-CMV-shRNA for shRNA-ETV1 construction and pLV-LAPTM4B vector for Lv-LAPTM4B construction) and two packaging plasmids, pH1 and pH2, were co-transfected into HEK-293T cells at a ratio of 0.5:0.35:0.15 for lentivirus production.

### Mouse tumor model

To establish a humanized immunodeficient mouse model, Huh7-LAPTM4B-Lv cells (2×10^6) were injected into BABL/c mice, initiating xenograft HCC. Immune cell depletion was achieved using Asialo GM1 antibody via tail vein injection, followed by human PBMC reconstitution after 7 days. Tumor size was monitored weekly until euthanasia at 28 days. Tumors were harvested for immune cell isolation and flow cytometry analysis.

### Western-blotting

Cold lysis buffer (Solarbio Life Sciences) was used to homogenize tumor tissues or cell pellets. The homogenate was centrifugated for 30 min at 8000 × g. After BCA quantification, supernatants were separated using SDS-PAGE gel and transferred onto a PVDF membrane. The membrane was incubated with anti-LAPTM4B, anti-ETV1, anti-c-Myc, anti-β-catenin, anti-Wnt3A, anti-Wnt1, anti-CXCL8, and anti-GAPDH antibodies (1:1000), washed with 1x TBST and incubated with HRP-conjugated secondary antibodies (1:2000). Following another wash with 1x TBST, the membrane was incubated with 1 ml chemiluminescent solution. Protein bands were visualized using Tanon imaging system. The sources of all antibodies can be found in Supplementary Table 1. All full-length western blots can be obtained in the supplementary materials.

### Total RNA extraction

Tissues were homogenized with TRIzol reagent and centrifuged at 3000 × g to remove pellets. Supernatants were mixed with chloroform, vortexed, and centrifuged at 12,000 × g. After transferring upper aqueous phase, RNA was precipitated with isopropanol (pelleted at 12,000 × g), washed with 70% ethanol, and collected after centrifugation at 8000 × *g*. The RNA was dissolved in RNase-free water.

### cDNA synthesis

cDNA was synthesized using SuperScript® III First-Strand Synthesis kit for RT-qPCR. Total RNA (2.5 µg) was combined with a 1 mM dNTP mix and 5 ng/µl random hexamer primers, and diluted with water to 5 µl. A mixture of RT buffer, RNaseOUT (2 U), DDT (10 mM), SuperScript® III (10 U), and MgCl2 (5 mM) was prepared in a 10 µl system. cDNA was synthesized following standard cDNA synthesis program and stored at −20 °C.

### Quantitative RT-PCR analysis

After mixing with the synthesized cDNA, the Sybr Green primer mix was briefly centrifuged. Then, 3.8 μl of ddH2O, 1 μl of cDNA, 5 μl of Sybr Green, and 0.2 μl of primers (10 μM) were added to a 384-well PCR plate. The PCR program conditions were: initial denaturation at 95 °C for 15 s, annealing at 56 °C for 30 s, and extension at 72 °C for 50 s, with totally 40 cycles. GAPDH was utilized as the reference gene.

### Statistical analysis

Cell clusters were identified using FindClusters function (resolution, 0.5) and annotated using marker genes from Human Cell Atlas (http://biocc.hrbmu.edu.cn/CellMarker/). RNA-Seq data from tumor and adjacent tissues were obtained from The Cancer Genome Atlas (TCGA). Pathway analysis was conducted on marker genes by Gene Set Enrichment Analysis (GSEA).

Statistical analysis was performed using R (version 4.2.3). Inter-variable differences were assessed using two-tailed Student’s t-tests. Progression-free survival (PFS), disease-free survival (DFS), overall survival (OS), and disease interval survival (DIS) were analyzed by log-rank tests. Multivariable analysis was performed using stepwise Cox proportional hazards regression models. P < 0.05 indicated statistical significance. Data were expressed as means±standard deviations.

## Results

### LAPTM4B up-regulation promoted immune cell infiltration in pan-cancer

**Fig. 1:**
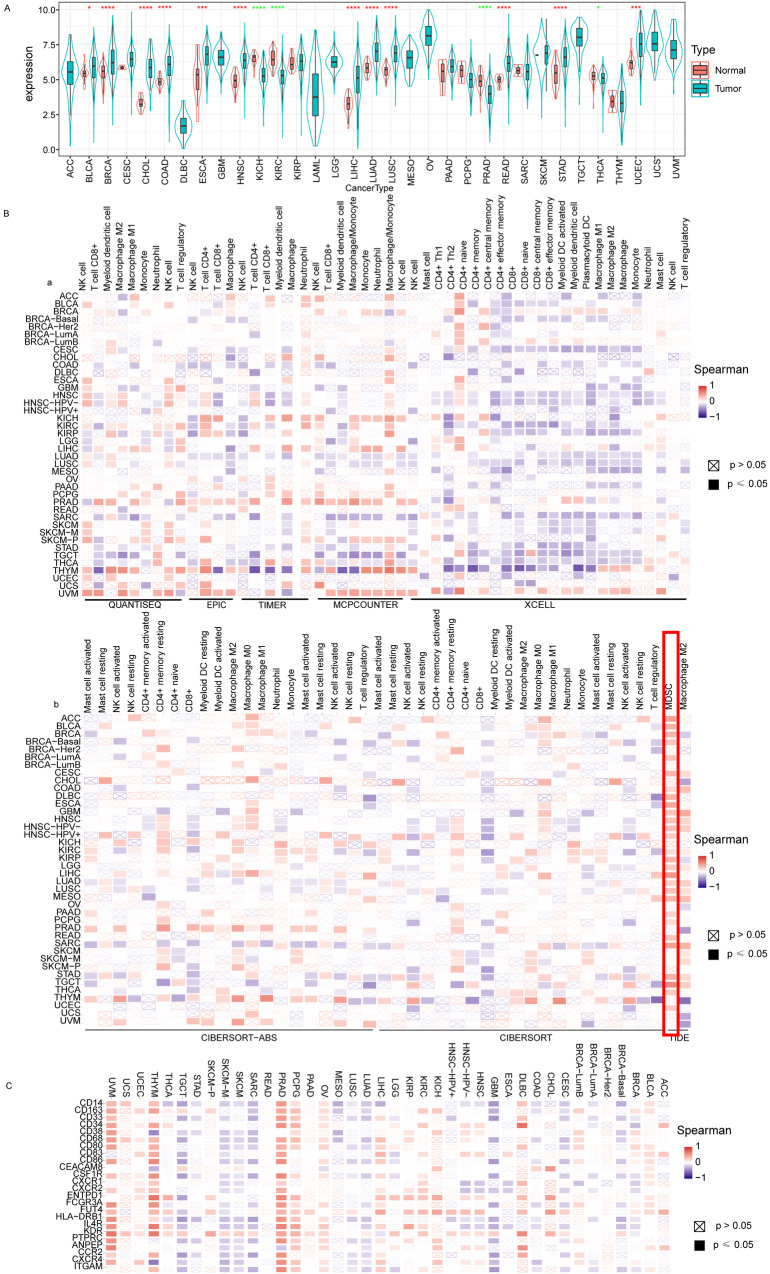
Expression of LAPTM4B and immune cell infiltration. **A** Differential expression of LAPTM4B in various tumor tissues and adjacent normal tissues. **B** Correlation between immune cell infiltration and LAPTM4B expression in tumors obtained through different computational methods. **C** The relationship between mdscs’ markers and LAPTM4B expression across different tumor types.

We acquired diverse tumor data from TCGA and identified LAPTM4B up-regulation in 12 of 33 tumor types, including Liver Hepatocellular Carcinoma (LIHC) (*p* < 0.0001) (Fig. 1A). We employed different algorithms to analyze immunological characteristics (Fig. 1B). MDSCs outperformed other immune cells, suggesting their pivotal role in LAPTM4B-mediated tumorigenesis (Fig. 1B, C).

### LAPTM4B up-regulation predicted pan-cancer prognosis

**Fig. 2:**
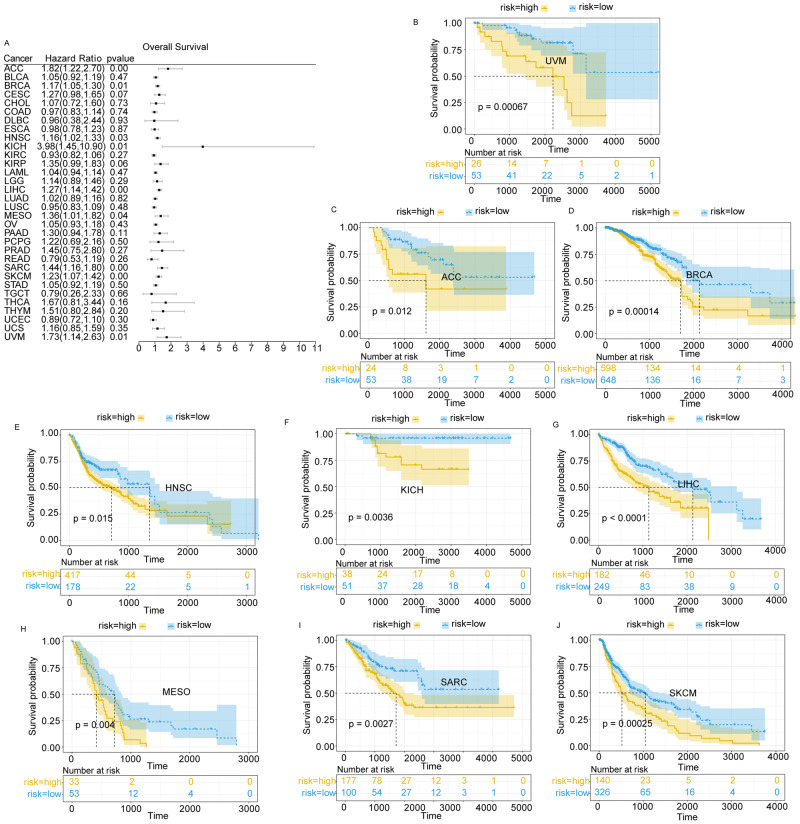
Impact of LAPTM4B expression on survival outcomes in different cancer patients. **A** Forest plot of overall survival in different tumor types. **B** Impact of high LAPTM4B expression on survival probability in uveal melanoma (UVM) patients. **C** Impact of high LAPTM4B expression on survival probability in adrenocortical carcinoma (ACC) patients. **D** Impact of high LAPTM4B expression on survival probability in breast cancer (BRCA) patients. **E** Impact of high LAPTM4B expression on survival probability in head and neck squamous cell carcinoma (HNSC) patients. **F** Impact of high LAPTM4B expression on survival probability in kidney chromophobe (KICH) patients. **G** Impact of high LAPTM4B expression on survival probability in liver hepatocellular carcinoma (LIHC) patients. **H** Impact of high LAPTM4B expression on survival probability in mesothelioma (MESO) patients. **I** Impact of high LAPTM4B expression on survival probability in sarcoma (SARC) patients. **J** Impact of high LAPTM4B expression on survival probability in skin cutaneous melanoma (SKCM) patients.

To evaluate how LAPTM4B expression affects cancer patient survival, we conducted survival analyses. The OS rates of 33 LAPTM4B-overexpressing cancers were evaluated (Fig. 2A). Kaplan-Meier survival curves were generated for cancer types including Uveal Melanoma (UVM), Adrenocortical Carcinoma (ACC), Breast Cancer (BRCA), Head and Neck Squamous Cell Carcinoma (HNSC), Kidney Chromophobe (KICH), LIHC, Mesothelioma (MESO), Sarcoma (SARC), and Skin Cutaneous Melanoma (SKCM) (Fig. 2B–J). Kaplan–Meier survival curves for LAPTM4B-overexpressing pan-cancer were examined for PFS, DFI, and DSS (Supplementary Figs. 1–3). LAPTM4B up-regulation had a detrimental influence on patient OS, especially in HCC.

### LAPTM4B up-regulation associated with aggressive clinicopathological characteristics and poor prognosis in HCC patients

**Fig. 3:**
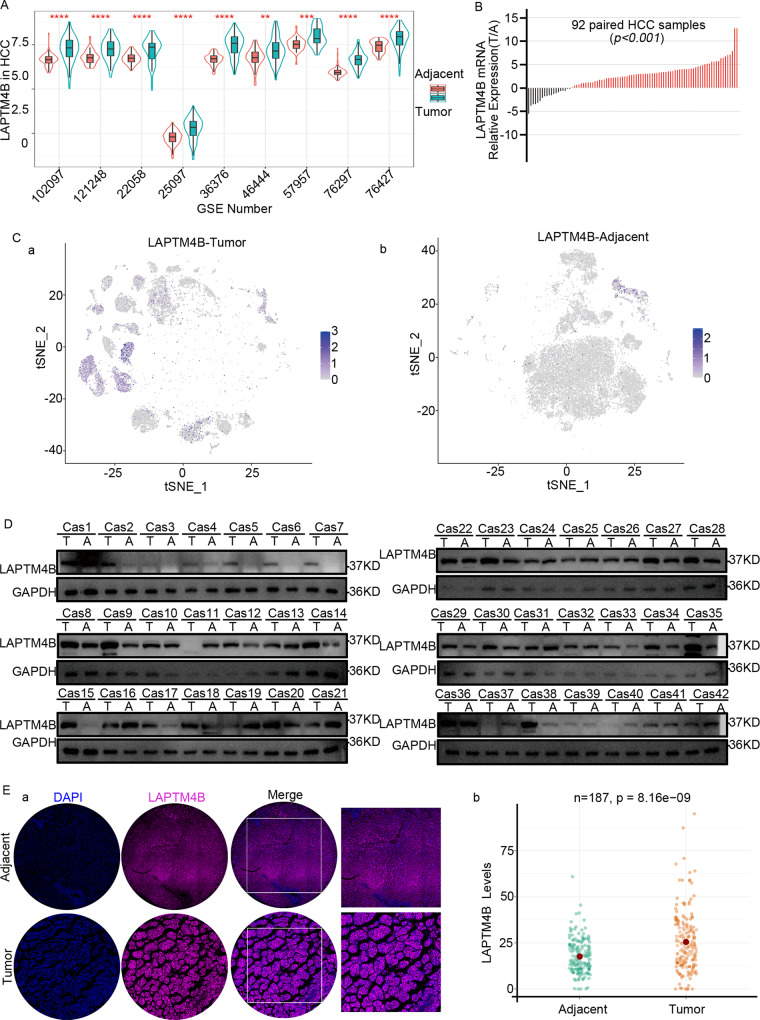
Expression of LAPTM4B in hepatocellular carcinoma and adjacent normal tissues. **A** LAPTM4B expression in hepatocellular carcinoma (HCC) tumors and adjacent tissues in the Gene Expression Omnibus (GEO). **B** Correlated Expression of LAPTM4B mRNA. **C** Single-cell sequencing results of LAPTM4B expression in tumor (a) and adjacent normal (b) tissues from TCGA database. **D** Western Blot Analysis of LAPTM4B Expression in 42 Pairs of HCC Tumor and Adjacent Tissues. **E** a. Immunofluorescence staining of LAPTM4B in HCC tumor and adjacent tissues; b. Dot plot representing LAPTM4B expression levels in tumor and adjacent tissues from 187 HCC patients at Eastern Hepatobiliary Surgery Hospital.

To mimic liver cancer development, a liver cancer mouse model was established. Mouse liver tissue immunohistochemistry revealed LAPTM4B up-regulation, which was associated with more MDSCs and cell adhesion molecules whereas fewer T cells (Supplementary Figure 4). Earlier survival analysis demonstrated an adverse impact of LAPTM4B up-regulation on four survival outcomes in HCC patients (Supplementary Fig. 5). To validate LAPTM4B expression in HCC, we examined LAPTM4B expression in HCC based on Gene Expression Omnibus (GEO) database. LAPTM4B expression significantly increased in HCC tissues compared with non-carcinoma tissues (Fig. 3A). PCR assays were performed on 92 paired samples of HCC tumor and adjacent normal tissues from The Affiliated Hospital of Qingdao University for validation; thus, LAPTM4B expression significantly increased in HCC tissues (*P* < 0.001; Fig. 3B).

These observations were verified through single-cell sequencing. LAPTM4B-positive cells were significantly accumulated in HCC tissues (Fig. 3Ca), but not in non-carcinoma tissues (Fig. 3Cb). LAPTM4B protein expression was verified on samples in 42 patients from The Affiliated Hospital of Qingdao University by Western-blotting assay, and LAPTM4B expression was significantly elevated in tumor tissues (Fig. 3D).

Tissue microarrays comprising 187 tissue samples from Eastern Hepatobiliary Surgery Hospital Affiliated to Naval Medical University were constructed. LAPTM4B expression was significantly different between tumor and normal tissues (*p* = 8.16e-09; Fig. 3E). Clinical and follow-up data were analyzed to monitor patient survival status. Patients with AFP > 20 ng/ml, age>50, tumor diameter>6 cm, early recurrence (+), and advanced BCLC stage exhibited LAPTM4B up-regulation (*P* < 0.05; Supplementary Fig. 6A). Therefore, LAPTM4B up-regulation predicted poorer clinical outcomes. Apart from LAPTM4B, variables like BMI and disease stage were unfavorable for patient OS and DFI (Supplementary Fig. 6B–E).

Therefore, LAPTM4B expression significantly increased in HCC tissues compared to non-carcinoma tissues, which predicted poor prognosis.

### ETV1 transcription activated LAPTM4B expression

**Fig. 4:**
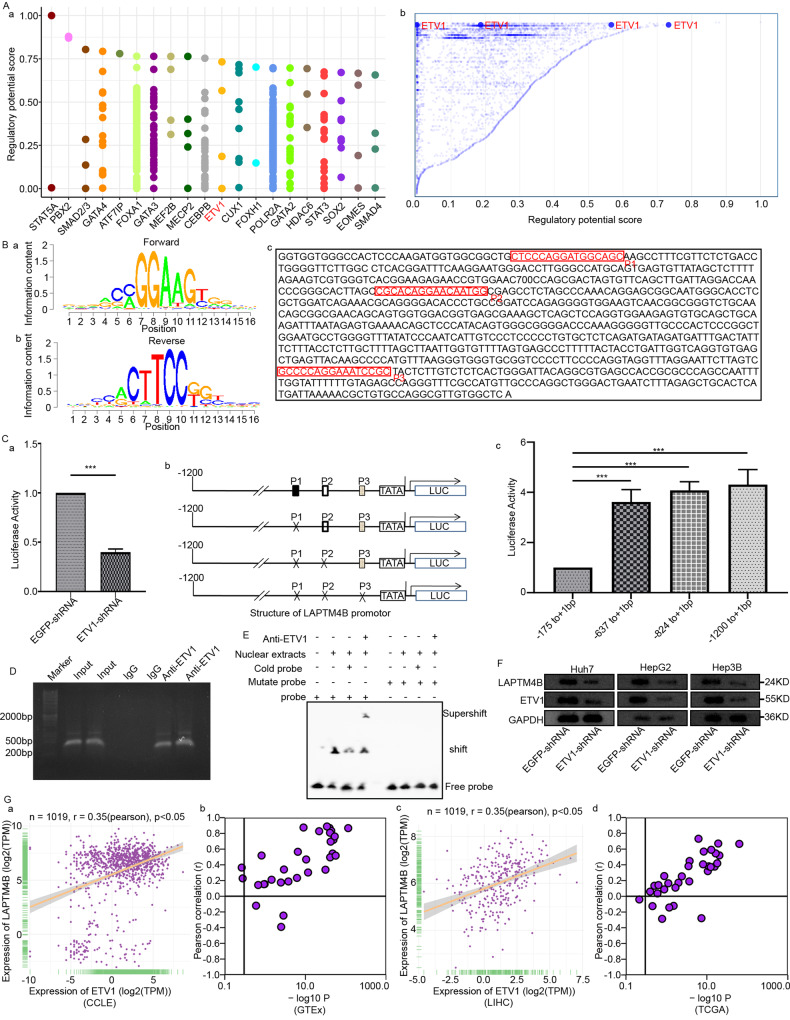
ETV1 binds to specific locations, leading to the transcription of LAPTM4B. **A** Data from the Cistrome DB database indicates that ETV1 is the primary transcriptional regulatory factor for LAPTM4B. **B** Gene expression motif plot highlighting the binding motif of ETV1 to the LAPTM4B promoter. **C** Dual-Luciferase reporter gene assay. a, Reduced activity of LAPTM4B after decreasing ETV1 expression with shRNA; b, Inhibition by sequences P1, P2, P3, depicted in the bar chart in c. **D** ETV1 binds to the endogenous promoter of LAPTM4B. Gene sequence analysis was performed to predict the positions of putative ETV1 binding sites within the LAPTM4B promoter, and primers for ChIP assays were designed. Chromatin from Huh7 cells was cross-linked, sonicated, and immunoprecipitated (IP) using ETV1 antibody or rabbit IgG. Specific primers targeting LAPTM4B promoter regions −842 bp to −809 bp, −637 bp to −622 bp, and −175 bp to −160 bp were used for qPCR to measure the promoter DNA quantity associated with IP chromatin. **E** ETV1 binds to the LAPTM4B promoter region −175 bp to −160 bp. Nuclear extracts were prepared from cells transfected with pHis-ETV1, and EMSA was performed using a FAM-labeled DNA probe synthesized from LAPTM4B promoter sequence −175 bp to −160 bp. Unlabeled probes (50x) or 1 μg of ETV1 antibody was added to the reaction to demonstrate the specificity of ETV1/DNA complex formation. EMSA was also conducted using a FAM-labeled mutated probe (Mut pro). **F** After silencing ETV1 using shRNA, Western blot experiments were conducted, and the expression of ETV1 and LAPTM4B decreased in Huh7, HepG2, and Hep3B cells. **G** The co-expression relationship between ETV1 and LAPTM4B was observed in CCLE, GTEx, LIHC, and TCGA datasets.

LAPTM4B expression depended on ETV1 in HCC (Fig. 4A). ETV1, also called ETS Related Protein 81 (ER81), belongs to Polyomavirus Enhancer Activator 3 (PEA3) subfamily (ETV1, ETV4, ETV5), with an N-terminal acidic transactivation domain [16]. ETV1 predicts HCC metastasis and poor prognosis [17].

To validate how ETV1 regulates LAPTM4B transcription, motifs and binding sequences were identified as P1-P3 (Fig. 4B). In dual-luciferase reporter gene assay, ETV1 activated −175 bp to −160 bp region of LAPTM4B promoter in Huh7 cells (Fig. 4C). Upon ChIP assays, ETV1 bound to −500 bp to −200 bp region of LAPTM4B promoter (Fig. 4D). EMSA indicated that FAM-labeled DNA probes synthesized from LAPTM4B promoter region formed DNA/protein complexes with ETV1. Adding unlabeled probes (50×) or ETV1-specific antibodies interfered with ETV1/DNA complex formation or formed supershifted complexes. However, FAM-labeled mutant probes could not form ETV1/DNA complexes (Fig. 4E).

Western-blotting was conducted for ETV1 and LAPTM4B expression in liver cancer cells. ETV1 knockdown decreased LAPTM4B expression (Fig. 4F), consistent with database-based validation results (Fig. 4G). Therefore, ETV1 was bound to a specific promoter region of LAPTM4B gene.

ETV1 was the potent transcription factor for LAPTM4B, which bound to LAPTM4B promoter region (-175bp to -160bp), facilitating LAPTM4B transcription.

### LAPTM4B promoted LCSCs through Wnt1/c-Myc/β-catenin Pathway

Tumor heterogeneity is caused by cells exhibiting characteristics similar to stem/progenitor cells, often called cancer stem cells (CSCs) [18]. Due to distinct stem cell-like self-renewal and differentiation abilities, CSCs regenerate distinctive tumor features. HCC tumor growth is driven by CSCs [19]. These LCSCs facilitate HCC initiation, progression metastasis, recurrence, and conventional chemotherapy and radiotherapy resistance [20].

**Fig. 5:**
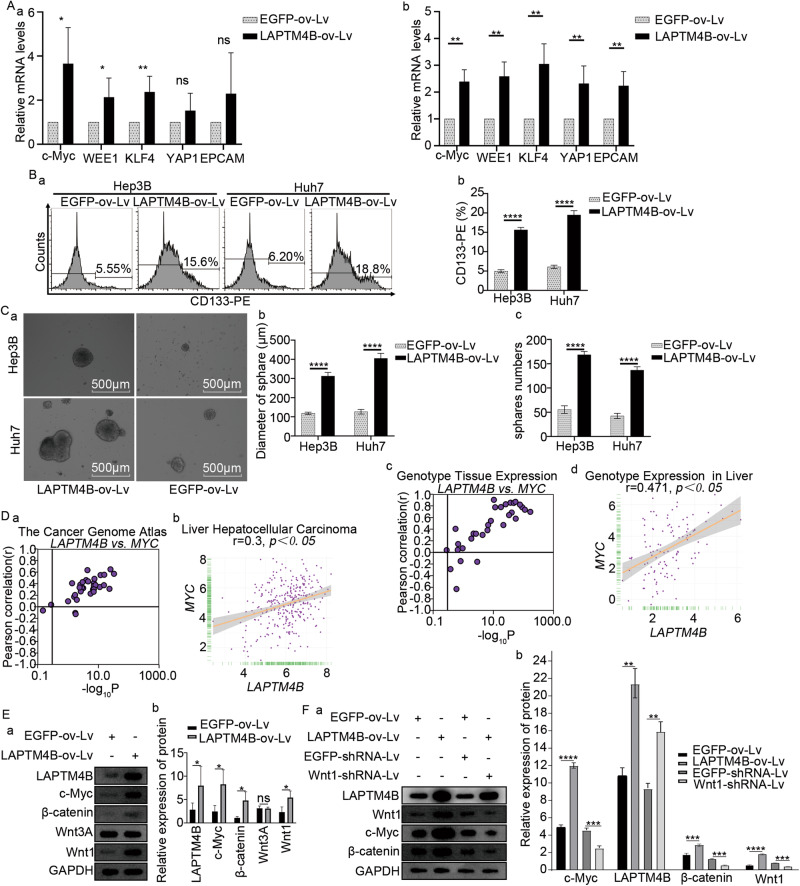
The regulatory role of LAPTM4B in hepatocellular carcinoma stem cells. **A** mRNA levels of hepatocellular carcinoma stem cell markers in Hep3B and Huh7 cell lines overexpressing LAPTM4B. **B** Flow cytometric analysis of CD133 expression in Hep3b and Huh7 cell lines overexpressing LAPTM4B. **C** Tumor spheroid assay: observation of the diameter and quantity of spheroids in Hep3B and Huh7 cell lines overexpressing LAPTM4B. **D** validation of the correlation between LAPTM4B and MYC expression in TCGA, LIHC, GTEx, and CCLE datasets. **E** construction of LAPTM4B overexpressing cell lines and validation of pathway-related protein expression. **F** Based on the results from E, silencing Wnt1 using shRNA resulted in a corresponding decrease in the expression levels of c-Myc and β-catenin.

To investigate the mechanism of LAPTM4B in LCSCs, LAPTM4B was over-expressed in Hep3B and Huh7 cells. PCR and flow cytometry revealed significant up-regulation of LCSC markers in LAPTM4B-overexpressing relative to control groups (Fig. 5A, B). Three-dimensional cell growth and tissue formation were explored by spheroid assay, yielding noteworthy results in cells. LAPTM4B overexpression increased tumor diameter and quantity (Fig. 5C).

To identify the specific pathways through which LAPTM4B induces LCSC proliferation, pathway enrichment analysis was conducted (Supplementary Fig. 7A, B). In HCC, significantly enriched pathways included E2F_TARGETS, G2M_CHECKPOINT, and MYC_TARGET, exhibiting enrichment concurrent with LAPTM4B up-regulation (Supplementary Fig. 7C–E; *p* < 2.2e-16). The association of MYC_TARGET with LAPTM4B expression was validated in a public database, revealing a robust correlation (Fig. 5D). LAPTM4B-overexpressing cells were established for Western-blotting, unveiling increased c-Myc, β-catenin, and Wnt1 phosphorylation levels in LAPTM4B-transfected cells (Fig. 5E). Wnt1 was silenced with shRNA, which down-regulated c-Myc and β-catenin (Fig. 5F). Therefore, LAPTM4B-induced expression in LCSCs via Wnt1/c-Myc/β-catenin pathway.

### LAPTM4B-induced MDSCs infiltration predicted poor prognosis

MDSCs are major immune suppressor cells primarily found under pathologies including chronic inflammation and cancer [21]. TME secretes various cytokines and chemokines to promote immature bone marrow cell generation and migration from bone marrow to the tumor site [22]. Human M-MDSCs are CD11b^+^ CD14^+^ CD33^+^ HLA-DR^low^, G-MDSCs are CD11b^+^ CD15^+^ CD66b^+^ HLA-DR^low^, while their murine counterparts are CD11b^+^ Ly6C^+^ and CD11b^+^ Ly6G^+^ Ly6C^low^, respectively [23].

**Fig. 6:**
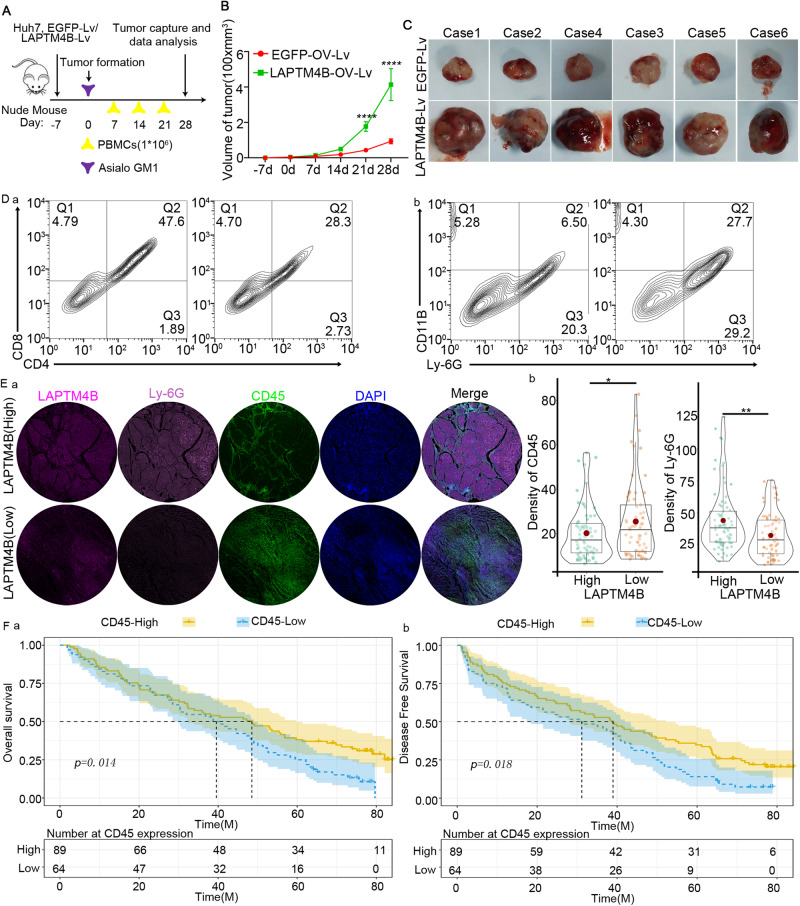
High expression of LAPTM4B induces MDSCs in tumor tissues. **A** Schematic Representation of the Construction Model for Humanized Immunodeficient Mice: Starting from the day of tumor formation, intravenous tail injection of Asialo GM1 antibody was performed to deplete immune cells. Human peripheral blood mononuclear cells (PBMCs) were injected on days 7, 14, 21, and 28. Tumor specimens were collected for analysis on day 28. **B** Line Graph Showing Tumor Volume Changes in LAPTM4B Overexpressing Tumors Compared to the Control Group from Day −7 to Day 28. **C** Photographs illustrating tumor volume changes in LAPTM4B overexpressing group compared to the control group. **D** Flow Cytometric Analysis of CD4+ and CD8+ Cells in Hepatic Tissues of Immunodeficient Mice. Compared to the control group (left panel in a), the immunodeficient group (right panel in a) exhibited decreased expression of CD4+ and CD8+ cells. Flow cytometric analysis of MDSCs in mice. Compared to the control group (left panel in b), the immunodeficient group (right panel in b) showed elevated levels of MDSCs. **E** Tissue microarray staining shows decreased cd45 density and increased ly-6g density in high LAPTM4B expression group compared to low expression group. **F** Survival KM curve illustrates Overall Survival and Disease-Free Survival of patients with high and low CD45 expression.

We introduced Asialo GM1 antibody in mice through tail vein injection to deplete immune cells, creating humanized immunodeficient mice that mimicked human immune system. Flow cytometry confirmed successful immunodeficient mouse construction, as evidenced by decreased CD4+/CD8+T cell expression (Fig. 6Da). Immunodeficient BABL/c mice were subcutaneously injected with 5*10^5 LAPTM4B-Lv and EGFP-Lv-infected Huh7 cells, and then with 1 × 10^6^ human PBMCs for immune system reconstitution (Fig. 6A). The tumor growth size was recorded from 0-28 days, and we observed a significant change in tumor volume after 21 days (Fig. 6B; *p* < 0.0001). Tumors were removed and examined on day 28, revealing increased LAPTM4B-Lv tumor size (Fig. 6C). MDSCs expression increased in TME of LAPTM4B-Lv (Fig. 6Db).

We stained tissue microarrays of patients from Eastern Hepatobiliary Surgery Hospital. LAPTM4B-overexpressing patients showed decreased T cell marker CD45 expression and increased MDSCs marker Ly-6G expression (Fig. 6E). Upon follow-up analysis, CD45 down-regulation predicted reduced OS and DFS (Fig. 6F). LAPTM4B up-regulation induced MDSCs migration, suppressed immune cell function, and adversely affected patient survival.

### LAPTM4B activated a suppressed TME via CXCL8 to promote MDSCs infiltration

**Fig. 7:**
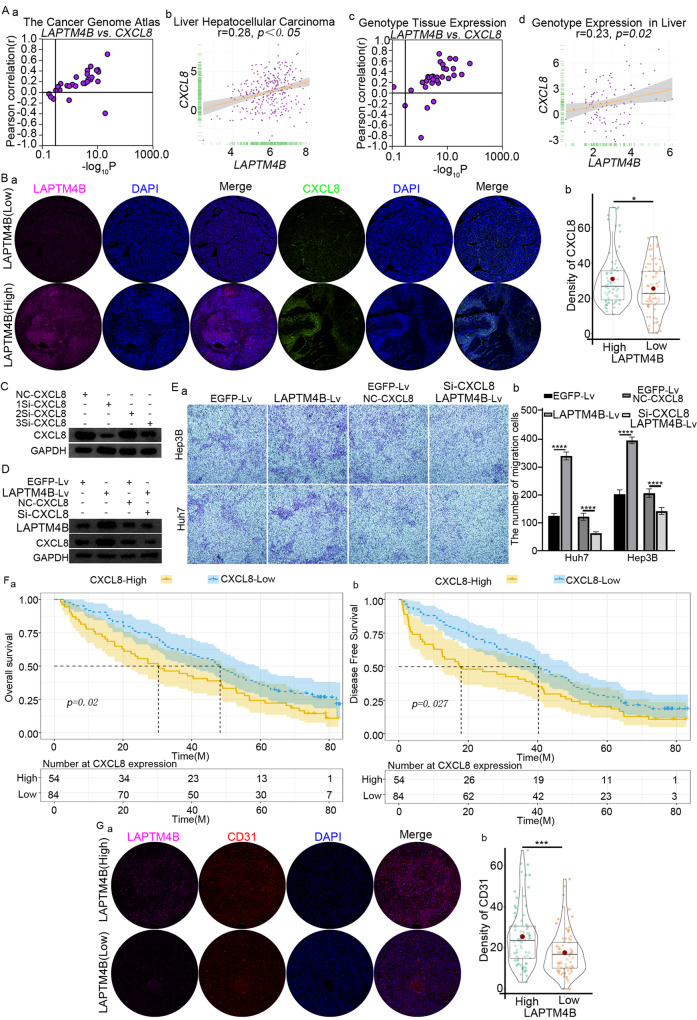
CXCL8 drives migration of MDSCs. **A** Co-expression correlation of CXCL8 and LAPTM4B across TCGA, LIHC, GTEx, and CCLE datasets. **B** Correlation between high and low expression of LAPTM4B and CXCL8 observed through tissue microarray staining (a) and visualized using violin plots (b). **C** Western blot experiments confirming the impact of siRNA interference targeting CXCL8 at varying transfection efficiencies. **D** Based on the results from **C**, selected transfection efficiencies were used to validate the correlation between LAPTM4B and CXCL8 expressions through Western blotting. **E** Migration experiments of MDSCs were conducted in both Huh7 and Hep3B cell lines using Transwell assays. The results were visualized through bar charts. **F** KM survival curves were generated for overall survival (OS) and disease-free survival (DFS) using data from 138 patients from the Eastern Hepatobiliary Hospital. The study aimed to observe the survival rates of patients with high and low expression levels of CXCL8. **G** Tissue microarray staining was performed to observe the correlation between high and low expression of LAPTM4B in patients and CD31 (a). The results were visualized using violin plots (b).

To substantiate the molecular mechanism of LAPTM4B in inducing MDSC migration, four different databases were analyzed, suggesting CXCL8 as a candidate cytokine regulated by LAPTM4B (Fig. 7A). CXCL8, also called interleukin-8 (IL-8), is a multifunctional chemokine, regulating tumor proliferation, invasion, and migration, often via autocrine or paracrine pathways [24]. Tissue microarray analysis revealed CXCL8 overexpression in TME of LAPTM4B-overexpressing patients (Fig. 7B; *p* < 0.05).

siRNAs with varying interference efficiencies were designed to silence CXCL8. 1Si-CXCL8 demonstrated the highest silencing efficiency (Fig. 7C), and was used for CXCL8-targeted investigation. LAPTM4B overexpression increased CXCL8 expression, promoting MDSC migration (Fig. 7D, E). CXCL8 silencing suppressed MDSCs migration compared with baseline, even after LAPTM4B overexpression (Fig. 7E). Therefore, LAPTM4B drove MDSCs migration toward tumor tissue primarily via CXCL8.

We conducted longitudinal follows-up of patients and tissue chip staining analyses. CXCL8-overexpressing patients had reduced OS and DFS (Fig. 7F). LAPTM4B up-regulation significantly increased CD31 density (*p* < 0.001; Fig. 7G). CD31, a 130 kDa membrane glycoprotein, is in immunoglobulin superfamily and instrumental in mediating homophilic/heterophilic adhesion. It is primarily localized at intercellular junctions of endothelial cells [25]. Therefore, LAPTM4B up-regulation might promote tumor angiogenesis. Thus, LAPTM4B-secreted CXCL8 drove MDSCs migration into tumor tissues.

### PD-L1 antibody counteracted LAPTM4B-mediated HCC progression

PD-1, an immune checkpoint molecule on T cell surface, and its counterpart, PD-L1 (CD274) often overexpressed on cancer cell surface, form a binding interaction, which suppresses T cell proliferation and activation [26]. PD-1/PD-L1 pathway is vital for cancer immunotherapy, and targeting inhibitors make significant breakthroughs in treatment [27].

**Fig. 8:**
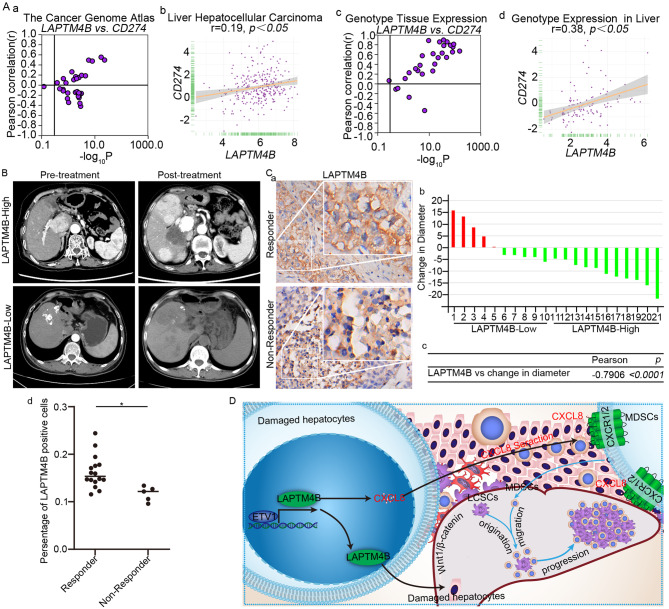
Correlation between LAPTM4B expression and Anti-PD-L1 therapy. **A** Co-expression correlation of CD274 and LAPTM4B across TCGA, LIHC, GTEx, and CCLE datasets. **B** Impact of LAPTM4B expression on PD-L1 antibody therapy, sourced from MRI data of patients at the Affiliated Hospital of Qingdao University. **C** Immunohistochemistry of LAPTM4B expression in patients responding and not responding to PD-L1 antibody therapy (a), followed by visualization of immunohistochemistry results (b, c, d). **D** ETV1 binds to the promoter region of LAPTM4B, inducing LAPTM4B transcription, promoting the proliferation of liver cancer stem cells (LCSCs) through the Wnt1/c-Myc/β-catenin pathway. LAPTM4B secretes the cytokine CXCL8, inducing the migration of myeloid-derived suppressor cells (MDSCs), leading to tumor progression.

Through public database analysis, LAPTM4B expression was strongly positively correlated with CD274 (Fig. 8A). In LAPTM4B-overexpressing patients, CD274 (PD-L1) overexpression on tumor surface suggests that treatment with PD-L1 antibodies may have therapeutic efficacy. We collected intraoperative samples and radiological data from 21 HCC patients undergoing PD-L1 therapy at The Affiliated Hospital of Qingdao University. LAPTM4B-overexpressing patients were responsive to PD-L1 therapy. Following PD-L1 treatment, tumor size significantly decreased (Fig. 8B). Immunohistochemistry revealed that, patients responding effectively to PD-L1 therapy exhibited LAPTM4B up-regulation (Fig. 8C; Pearson *r* = −0.7906, *p* < 0.0001).

LAPTM4B up-regulation substantially worsened HCC patient prognosis. However, these patients are responsive to PD-L1 antibody therapy, highlighting sensitivity of LAPTM4B-overexpressing patients to targeted treatments and underscoring effectiveness of PD-L1 blockade on mitigating LAPTM4B overexpression-related effects in HCC.

## Discussion

Immune-based therapies revolutionize systemic treatment of advanced cancer [28]. Applying ICIs, especially monoclonal antibodies targeting PD-1, PD-L1, and cytotoxic T-lymphocyte-associated protein 4 (CTLA-4), and modulating immune checkpoints on tumor cells, bring significant breakthroughs in treating solid tumors, including HCC [29, 30]. Immunotherapy is promising yet challenging for HCC treatment, due to highly unique liver immunological landscape with abundant immune cells. We elucidated the mechanism of LAPTM4B in inducing HCC development and demonstrated its potential to guide PD-L1 mAb therapy (Fig. 8D).

LAPTM4B is a susceptibility gene in cancers, including HCC [31]. To explore its regulatory mechanisms in HCC, we identified ETV1, which was bound to sequences located approximately 200 base pairs upstream of LAPTM4B promoter. ETV1 is an oncogenic driver in cancers, like prostate cancer and Ewing’s sarcoma [32, 33]. However, its interaction with LAPTM4B in HCC represents a novel area of investigation. LAPTM4B promoted LCSC proliferation. LCSCs exist in premalignant liver regions and contain altered liver cell foci showing CSC-like characteristics [18]. Therefore, LCSCs-LAPTM4B interaction becomes crucial for HCC development. LAPTM4B-mediated LSCS expression via Wnt1/c-Myc/β-catenin pathway. Wnt1 can regulate cancer progression by promoting cancer cell proliferation, migration, and survival [34, 35]. WNT1 binds to Frizzled (FZD) receptors, activating intracellular pathways to promote β-catenin accumulation and nuclear localization [36].

LAPTM4B expression activates LCSCs proliferation and CXCL8 secretion, facilitating MDSCs migration. MDSCs induce tumor development and metastasis, exacerbating LCSC transformation into HCC. Cytokines and chemokines, especially the CXCL8-CXCR1/2 axis, are vital for promoting inflammation, tumor progression, and immunotherapy resistance [37, 38]. CXCL8-CXCR1/2 axis is related to neutrophils or MDSCs recruitment, and CXCL8 affects immune cell functions, impacting tumor development. LAPTM4B-produced CXCL8 attracts MDSCs to TME and triggers neutrophil MDSCs to extrude NETs, facilitating tumor cell nesting [39]. LAPTM4B exerted dual impacts on inducing LCSCs and MDSCs, explaining the poor prognosis in LAPTM4B-overexpressing patients (Supplementary Figure 6).

Clinical trials of PD-L1/PD-1 antibodies in HCC show promising results; however, the response rate (20%) is lower than immunogenic tumors like melanoma and Hodgkin lymphoma (40%-90%). The distinguishing feature is that most responsive patients show PD-L1 up-regulation, elevated intratumoral CD8^+^ T cell levels, and less immunosuppressive microenvironment [40–42]. Although LAPTM4B-mediated MDSCs aggregation to inhibit immune response, patients responded positively to PD-L1 antibody therapy. PD-L1 antibodies relieve inhibitory signals between PD-1 and PD-L1 to restore CD8^+^ T cell activity and help immune system target tumor cells. Thus, even immune-suppressive cells (MDSCs and TAMs) are present, PD-1 antibodies partially activate T cells and help immune system resist tumor. However, clinical efficacy varies due to individual differences and specific tumor characteristics [43, 44].

LAPTM4B is crucial for regulating tumor suppression, tumor cell proliferation, invasion, metastasis, apoptosis resistance, autophagy initiation, and drug resistance. The upstream promoter ETV1 and its binding sequence for LAPTM4B are identified. LAPTM4B overexpression facilitates LCSC proliferation, MDSC migration, and enhances anti-PD-L1 efficacy. These findings assist in molecular diagnostics and guide therapeutic strategies.

### Supplementary information

supplementary materials

Original Data

Antibody colonies used in this article

aj-checklist

Supplementary Figure1

Supplementary Figure2

Supplementary Figure3

Supplementary Figure4

Supplementary Figure5

Supplementary Figure6

Supplementary Figure7

Supplementary Figure annotation

## Data Availability

The data and material are available by contacting the corresponding author upon reasonable request.
