# Supplementary material for: LAPTM4B-mediated hepatocellular carcinoma stem cell proliferation and MDSC migration: implications for HCC progression and sensitivity to PD-L1 monoclonal antibody therapy
Source: Cell Death Dis. 2024 Feb 22;15(2):165. doi: 10.1038/s41419-024-06542-8 (PMC10884007; doi:10.1038/s41419-024-06542-8)
Supplement: Supplementary file 1 — supplementary materials [file 41419_2024_6542_MOESM1_ESM.pdf]

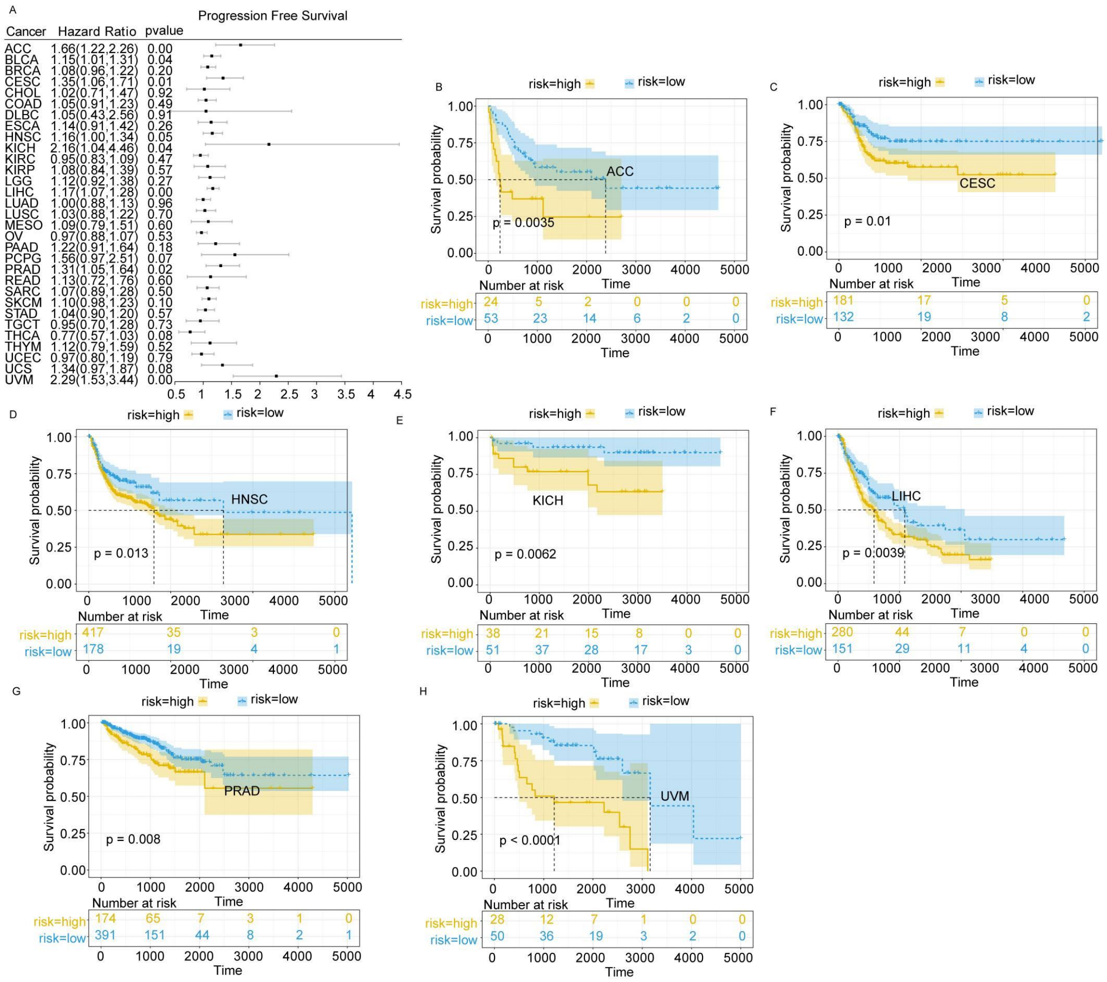

Supplementary Figure 1 Impact of LAPTM4B expression on Progression-Free Survival (PFS) in different cancer patients

A: Forest plot of overall survival in different tumor types.

B-H: Impact of high LAPTM4B expression on PFS in different tumors patients.

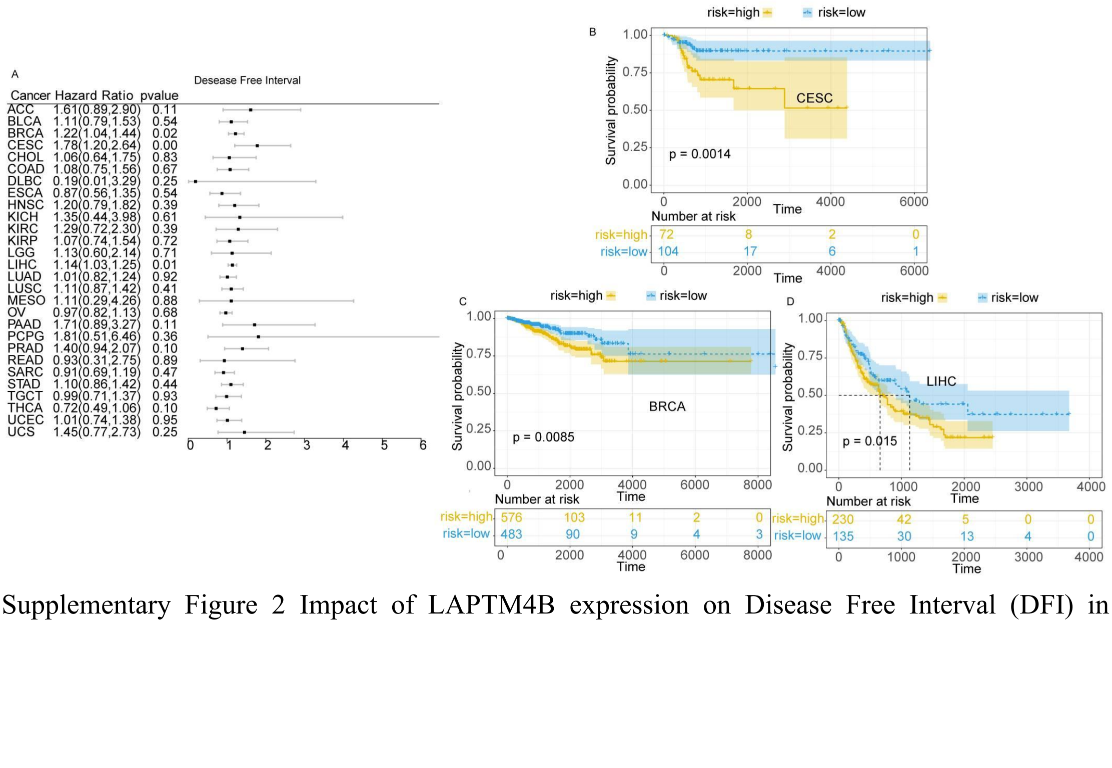

Supplementary Figure 2 Impact of LAPTM4B expression on Disease Free Interval (DFI) in

different cancer patients

A: Forest plot of DFI in different tumor types.

B-D: Impact of high LAPTM4B expression on DFI in different tumors patients.

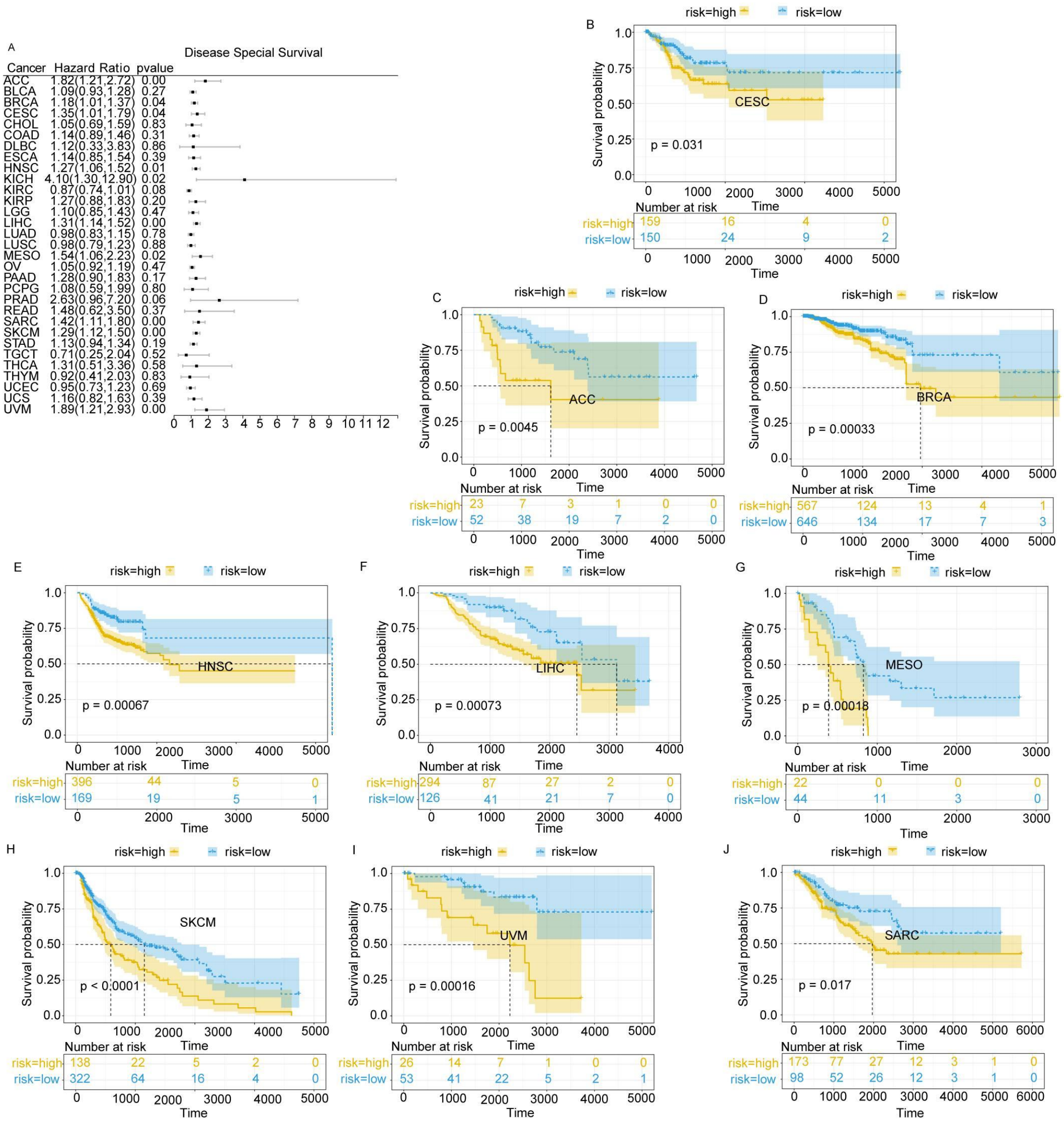

Supplementary Figure 3 Impact of LAPTM4B expression on Disease Specific Survival (DSS) in different cancer patients

A: Forest plot of DSS in different tumor types.

B-J: Impact of high LAPTM4B expression on DSS in different tumors patients.

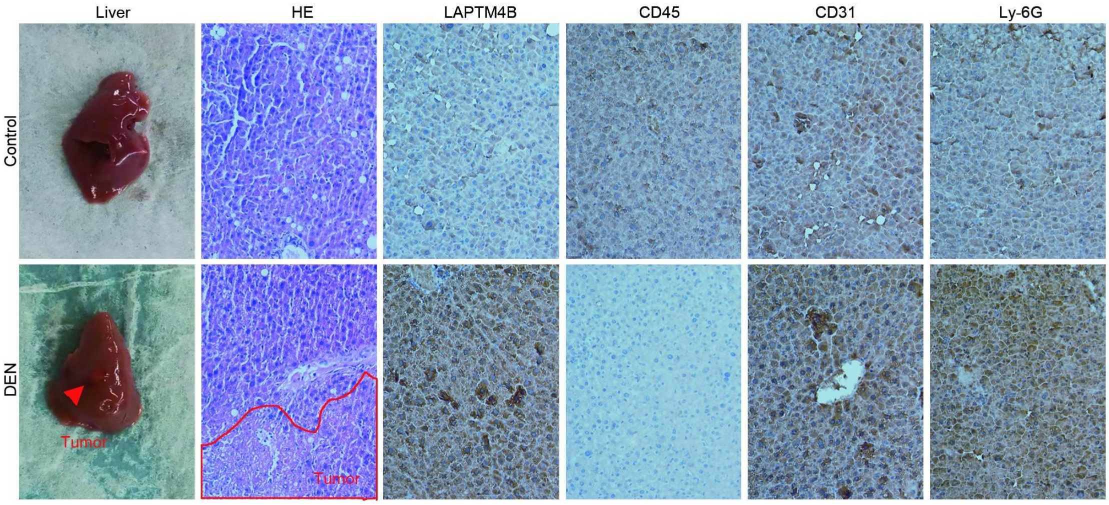

Supplementary Figure 4 Mouse hepatocellular carcinoma model

The model construction method can be found in the Methods section.Tissue specimens were subjected to Hematoxylin and Eosin (HE) staining and immunohistochemistry, followed by examination and imaging under a microscope.

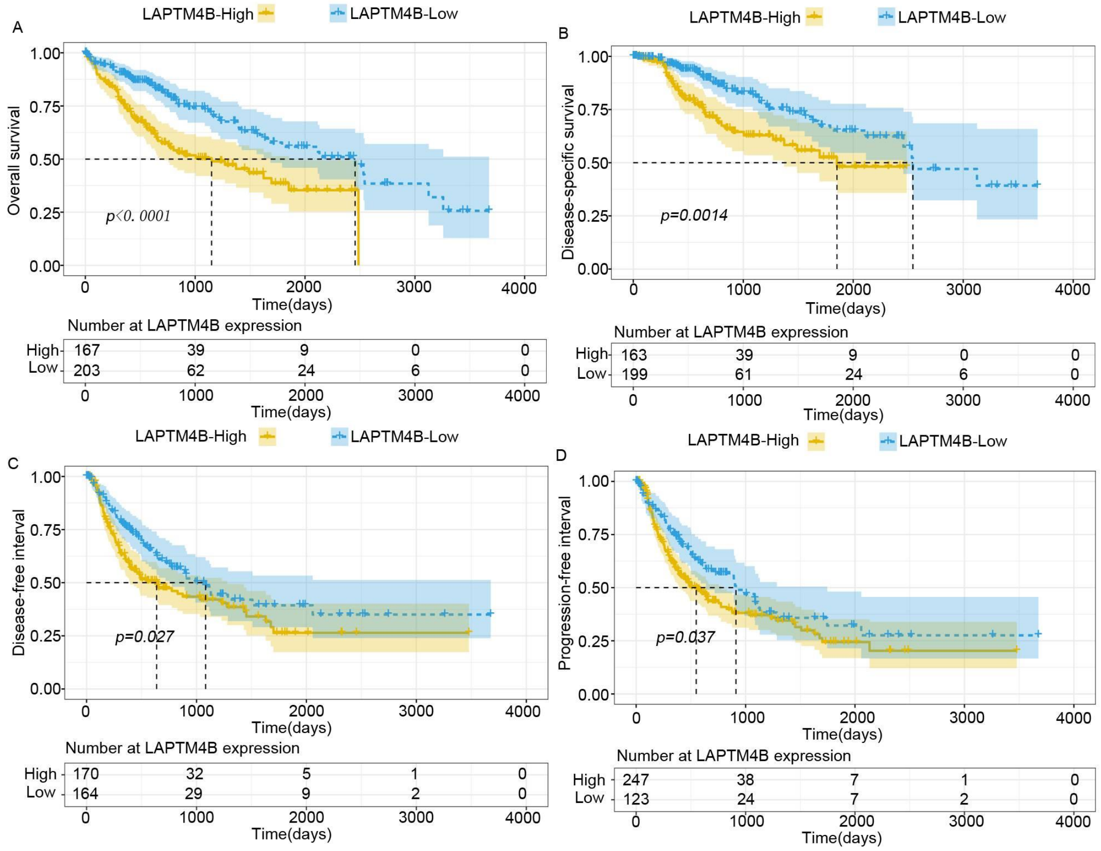

Supplementary Figure 5 Survival curves for patients with high and low LAPTM4B expression

A: The impact of LAPTM4B expression on overall survival (OS) of hepatocellular carcinoma (HCC) patients in the TCGA database.

B: The impact of LAPTM4B expression on Disease-Specific Survival (DSS) of hepatocellular carcinoma (HCC) patients in the TCGA database.

C: The impact of LAPTM4B expression on Disease-Free Interval (DFI) of hepatocellular carcinoma (HCC) patients in the TCGA database.

D: The impact of LAPTM4B expression on Progression-Free Interval （PFI） of hepatocellular carcinoma (HCC) patients in the TCGA database.

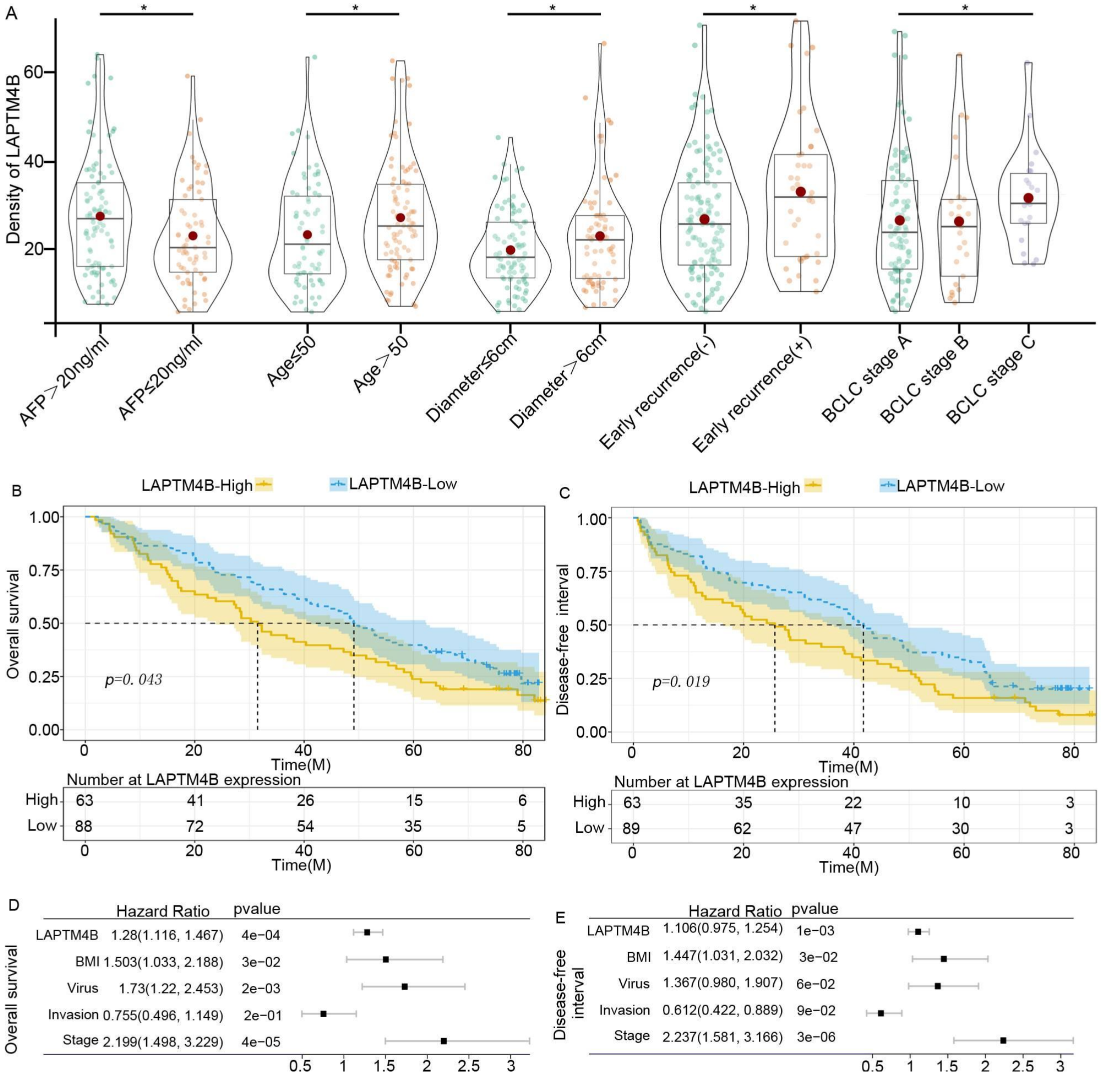

Supplementary Figure 6 Clinical information from patients at the Eastern Hepatobiliary Hospital

A: Violin plot showing the correlation between LAPTM4B density and clinical indicators in patients.

B: Overall survival (OS) curves for patients with high and low LAPTM4B expression.

C: Disease-Free Interval (DFI) curves for patients with high and low LAPTM4B expression.

D: Risk factors affecting patient Overall survival (OS).

E: Risk factors affecting patient Disease-Free Interval (DFI).

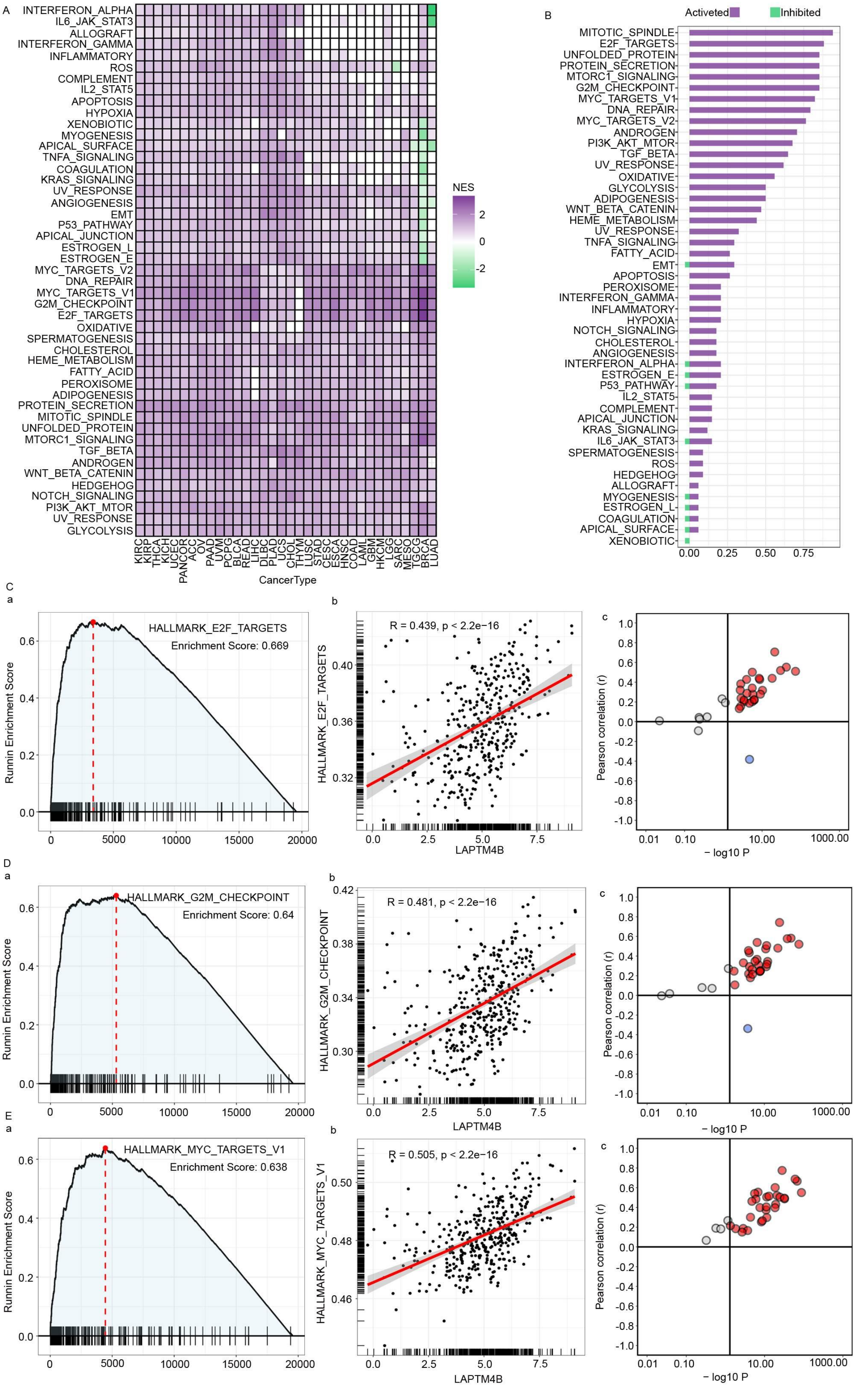
A
INTERFERON_ALPHA
IL6_JAK_STAT3
ALLOGRAFT
INTERFERON_GAMMA
INFLAMMATORY
ROS
COMPLEMENT
IL2_STAT5
APOPTOSIS
HYPOXIA
XENOBIOTIC
MYOGENESIS
APICAL_SURFACE
TNFA_SIGNALING
COAGULATION
KRAS_SIGNALING
UV_RESPONSE
ANGIOGENESIS
EMT
P53_PATHWAY
APICAL_JUNCTION
ESTROGEN_L
ESTROGEN_E
MYC_TARGETS_V2
DNA_REPAIR
MYC_TARGETS_V1
G2M_CHECKPOINT
E2F_TARGETS
OXIDATIVE
SPERMATOGENESIS
CHOLESTEROL
HEME_METABOLISM
FATTY_ACID
PEROXISOME
ADIPOGENESIS
PROTEIN_SECRETION
MITOTIC_SPINDLE
UNFOLDED_PROTEIN
MTORC1_SIGNALING
TGF_BETA
ANDROGEN
WNT_BETA_CATENIN
HEDGEHOG
NOTCH_SIGNALING
PI3K_AKT_MTOR
UV_RESPONSE
GLYCOLYSIS
KIRC KIRP THCA KICH UCEC PANCOR ACC OV PAAD UVM PCPG BLCA READ LIHC DLBC PLAD UCS CHOL THYM LUSC STAD CESC ESCA HNSC COAD LAML GBM HKCM LGG SARC MESO TGCG BRCA LUAD
CancerType
NES
2
0
-2
B
Activeted
Inhibited
MITOTIC_SPINDLE
E2F_TARGETS
UNFOLDED_PROTEIN
PROTEIN_SECRETION
MTORC1_SIGNALING
G2M_CHECKPOINT
MYC_TARGETS_V1
DNA_REPAIR
MYC_TARGETS_V2
ANDROGEN
PI3K_AKT_MTOR
TGF_BETA
UV_RESPONSE
OXIDATIVE
GLYCOLYSIS
ADIPOGENESIS
WNT_BETA_CATENIN
HEME_METABOLISM
UV_RESPONSE
TNFA_SIGNALING
FATTY_ACID
EMT
APOPTOSIS
PEROXISOME
INTERFERON_GAMMA
INFLAMMATORY
HYPOXIA
NOTCH_SIGNALING
CHOLESTEROL
ANGIOGENESIS
INTERFERON_ALPHA
ESTROGEN_E
P53_PATHWAY
IL2_STAT5
COMPLEMENT
APICAL_JUNCTION
KRAS_SIGNALING
IL6_JAK_STAT3
SPERMATOGENESIS
ROS
HEDGEHOG
ALLOGRAFT
MYOGENESIS
ESTROGEN_L
COAGULATION
APICAL_SURFACE
XENOBIOTIC
0.00 0.25 0.50 0.75
C
a
HALLMARK_E2F_TARGETS
Enrichment Score: 0.669
Runnin Enrichment Score
0.0 0.2 0.4 0.6
0 5000 10000 15000 20000
b
R = 0.439, p < 2.2e-16
HALLMARK_E2F_TARGETS
0.32 0.36 0.40
0.0 2.5 5.0 7.5
LAPTM4B
c
Pearson correlation (r)
1.0 0.8 0.6 0.4 0.2 0.0 -0.2 -0.4 -0.6 -0.8 -1.0
0.01 0.10 10.00 1000.00
− log10 P
D
a
HALLMARK_G2M_CHECKPOINT
Enrichment Score: 0.64
Runnin Enrichment Score
0.0 0.2 0.4 0.6
0 5000 10000 15000 20000
b
R = 0.481, p < 2.2e-16
HALLMARK_G2M_CHECKPOINT
0.30 0.34 0.38 0.42
0.0 2.5 5.0 7.5
LAPTM4B
c
Pearson correlation (r)
1.0 0.8 0.6 0.4 0.2 0.0 -0.2 -0.4 -0.6 -0.8 -1.0
0.01 0.10 10.00 1000.00
− log10 P
E
a
HALLMARK_MYC_TARGETS_V1
Enrichment Score: 0.638
Runnin Enrichment Score
0.0 0.2 0.4 0.6
0 5000 10000 15000 20000
b
R = 0.505, p < 2.2e-16
HALLMARK_MYC_TARGETS_V1
0.46 0.48 0.50
0.0 2.5 5.0 7.5
LAPTM4B
c
Pearson correlation (r)
1.0 0.8 0.6 0.4 0.2 0.0 -0.2 -0.4 -0.6 -0.8 -1.0
0.01 0.10 10.00 1000.00
− log10 P

Supplementary Figure 7 Metabolic pathways involved in LAPTM4B

A: Heatmap illustrating the enrichment of metabolic pathways involving LAPTM4B in pan-cancer contexts.

B: Bar chart depicting enriched metabolic pathways involving LAPTM4B in hepatocellular carcinoma (HCC).

C: Enrichment analysis of LAPTM4B with E2F_TARGETS.

D: Enrichment analysis of LAPTM4B with G2M_CHECKPOINT.

E: Enrichment analysis of LAPTM4B with MYC_TARGETS.

Supplementary Table 1. Antibody colonies used in this article

Antibody colonies	Source	Identifier
LAPTM4B Polyclonal antibody	Proteintech	18895-1-AP
Anti-ER81/ETV1 antibody	Abcam	ab314874
c-MYC Monoclonal antibody	Proteintech	67447-1-Ig
Beta Catenin Monoclonal antibody	Proteintech	66379-1-Ig
WNT3A Rabbit pAb	ABclonal	A0642
WNT1 Rabbit pAb	ABclonal	A2475
FITC-conjugated Goat Anti-Rabbit IgG	Elabscience	E-AB-1014
FITC-conjugated Goat Anti-Mouse IgG	Elabscience	E-AB-1015
TRITC-conjugated Goat Anti- Rabbit IgG	Elabscience	E-AB-1053
HRP-conjugated Goat Anti-Mouse IgG	Elabscience	E-AB-1008
HRP-conjugated Goat Anti-Rabbit IgG	Elabscience	E-AB-1003
Anti-Mouse Ly-6G (Gr-1) (RB6-8C5)	Proteintech	65140-1-Ig
GAPDH Monoclonal Antibody	Proteintech	60004-1-Ig
CoraLite® Plus 488 Anti-Mouse Ly-6G (1A8)	Proteintech	CL488-65078
PE Anti-Mouse CD11b (M1/70)	Proteintech	PE-65055
FITC Anti-Human CD4 (SK3)	Proteintech	FITC-65147
PE Anti-Human CD8a (RPA-T8)	Proteintech	PE-65144
CD45 Rabbit PolymAb® (A24802)	ABclonal	A24802
Recombinant Anti-IL-8 antibody[EPR26511-74]	Abcam	ab289992
Nuclear Extraction Kit	Abcam	ab113474
