# Supplementary figures and images for: LAPTM4B-mediated hepatocellular carcinoma stem cell proliferation and MDSC migration: implications for HCC progression and sensitivity to PD-L1 monoclonal antibody therapy
Source: Cell Death Dis. 2024 Feb 22;15(2):165. doi: 10.1038/s41419-024-06542-8 (PMC10884007; doi:10.1038/s41419-024-06542-8)

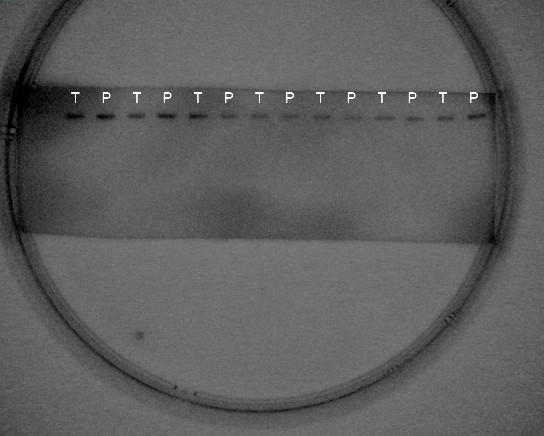

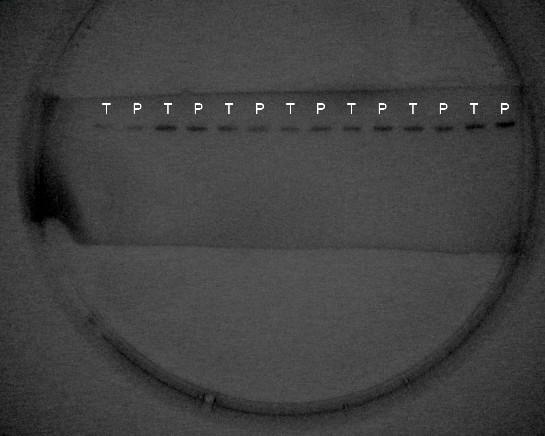

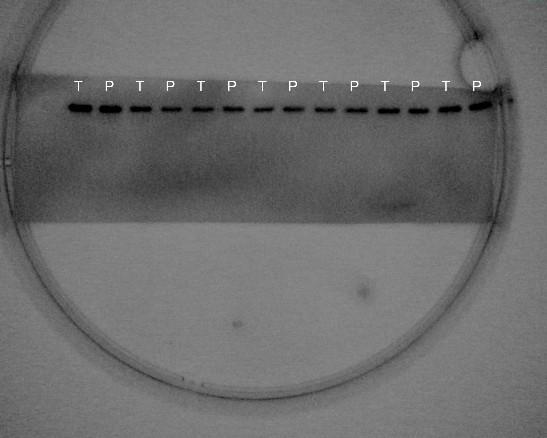

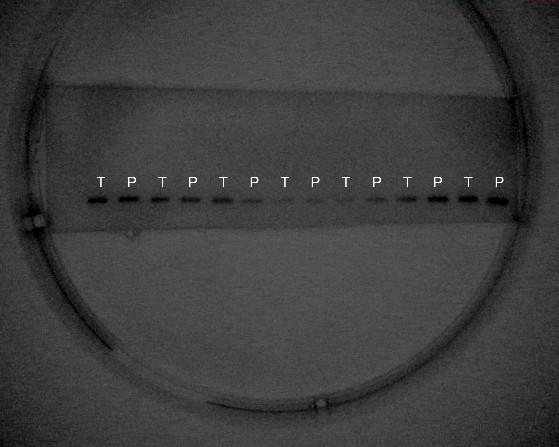

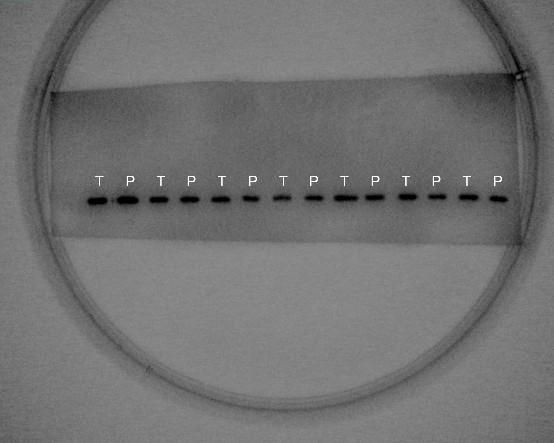

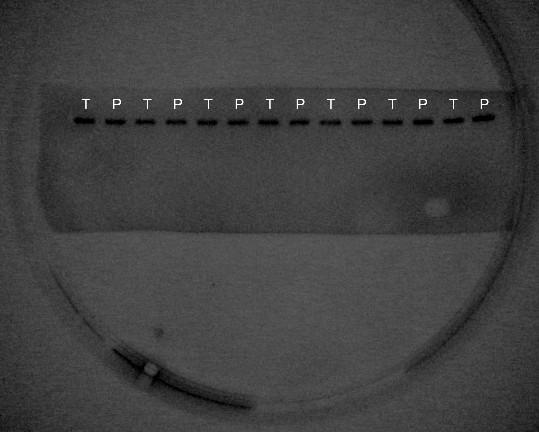


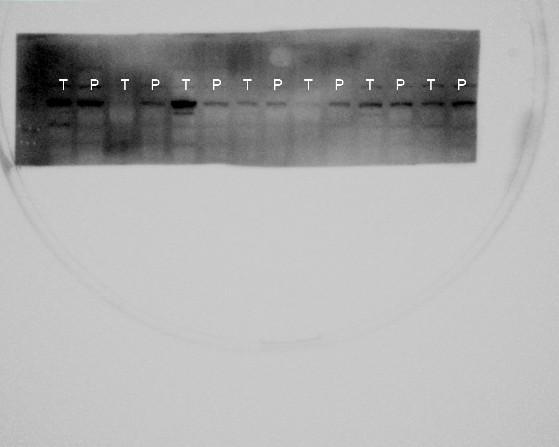

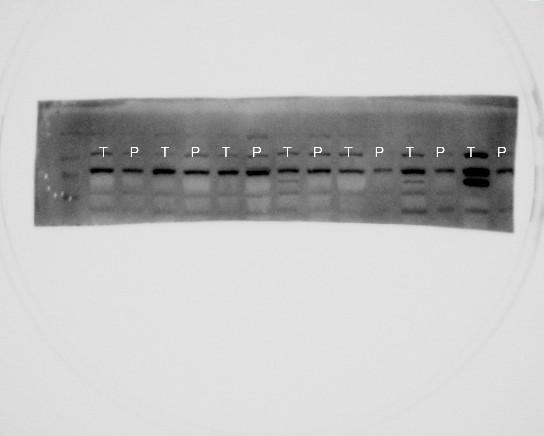

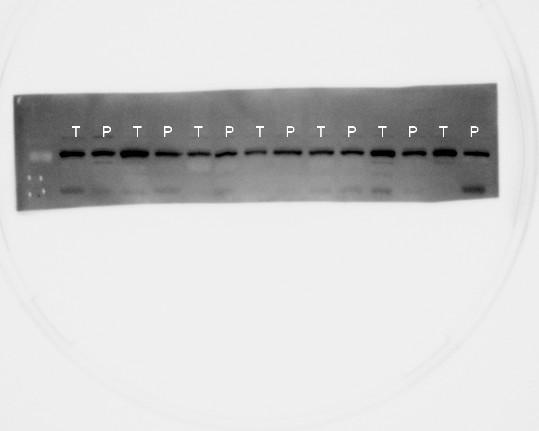

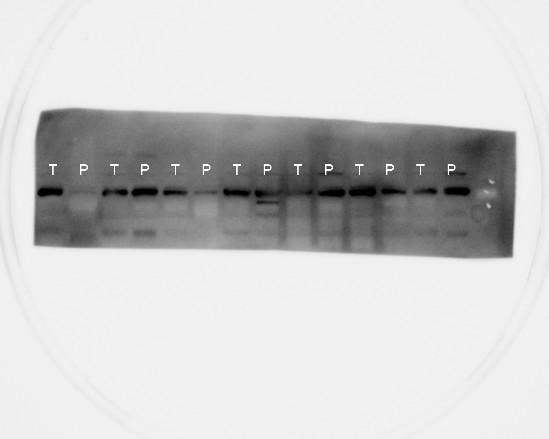

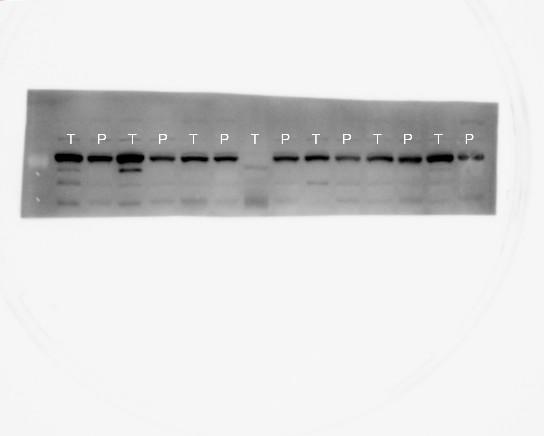

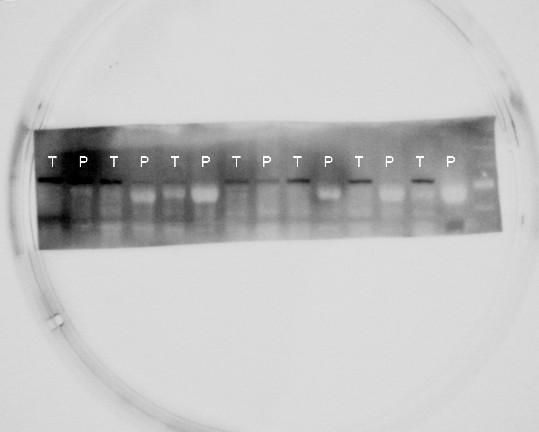


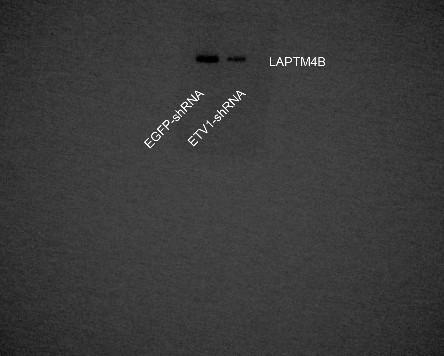

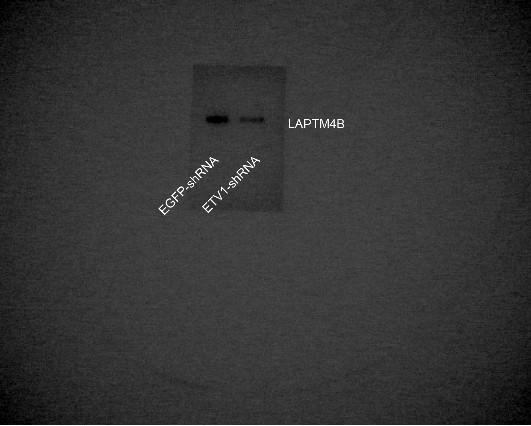

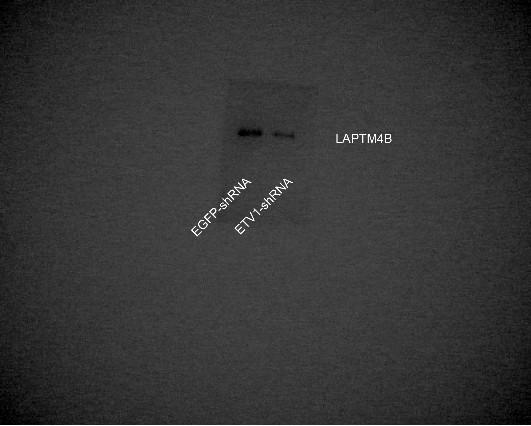

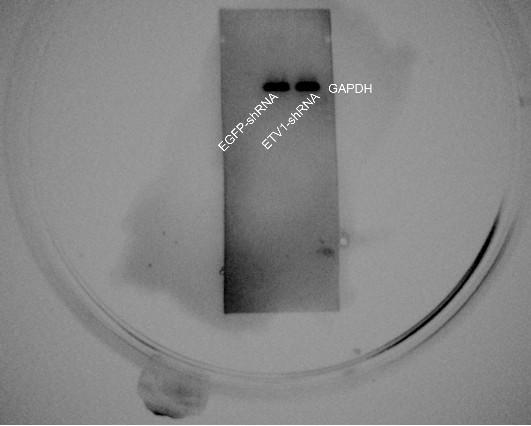

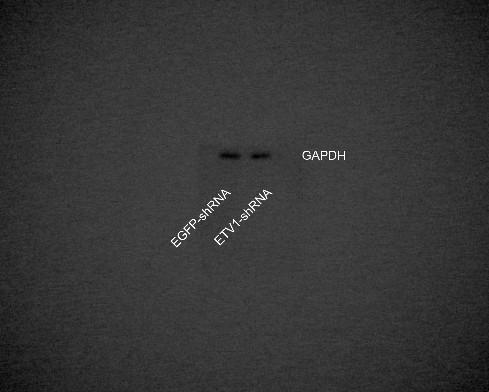

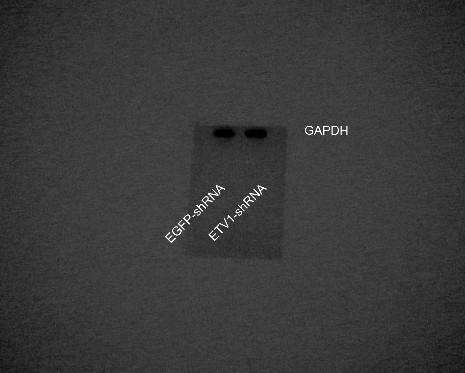

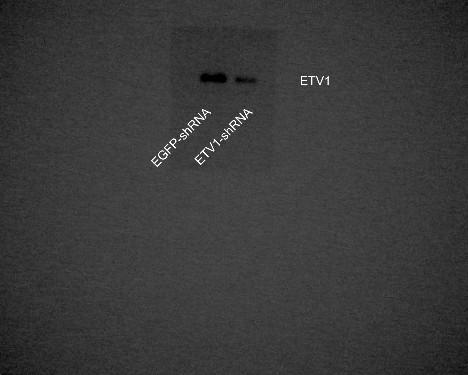

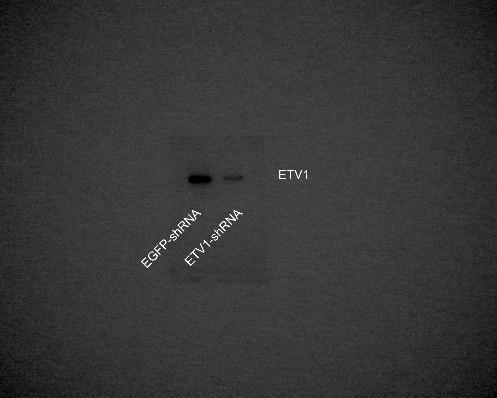

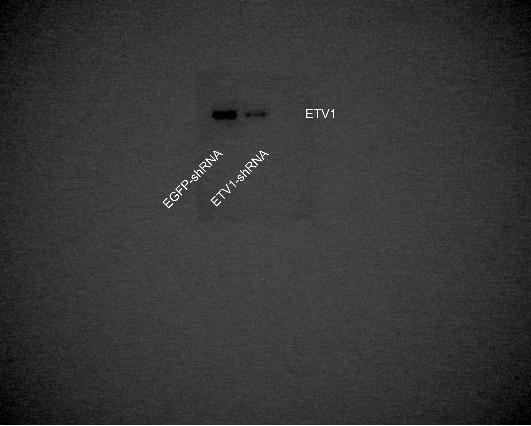

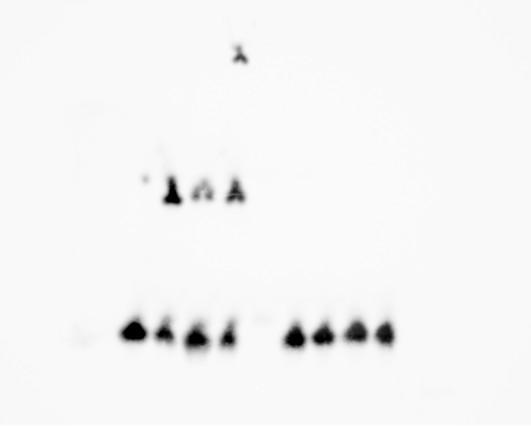

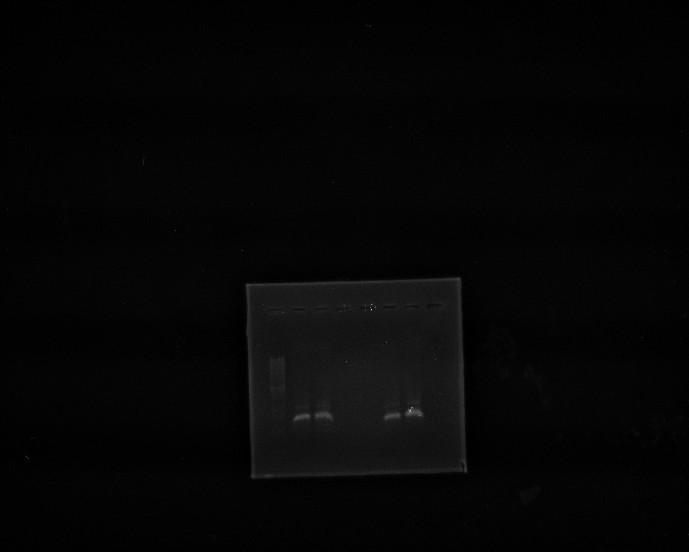


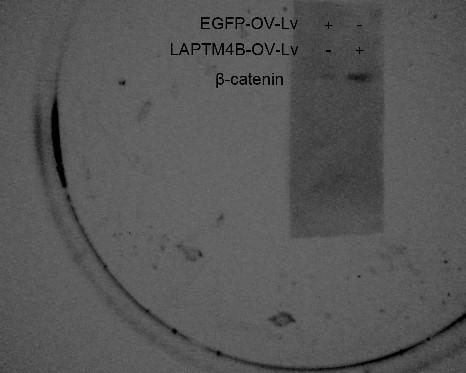

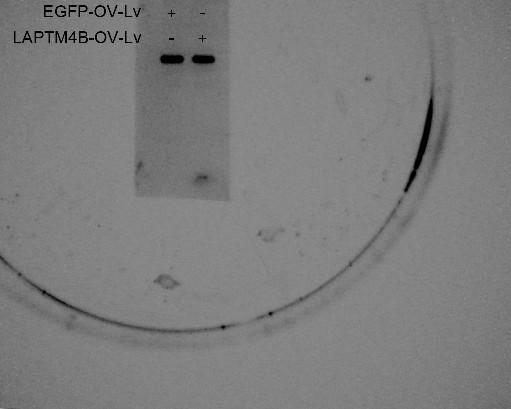

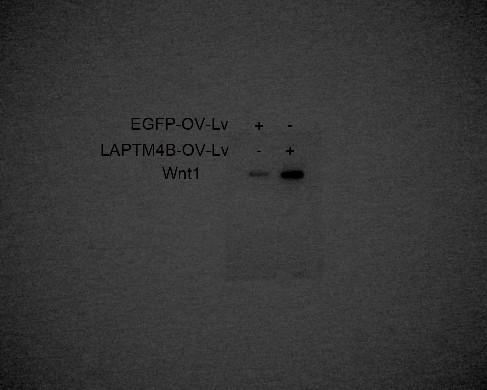

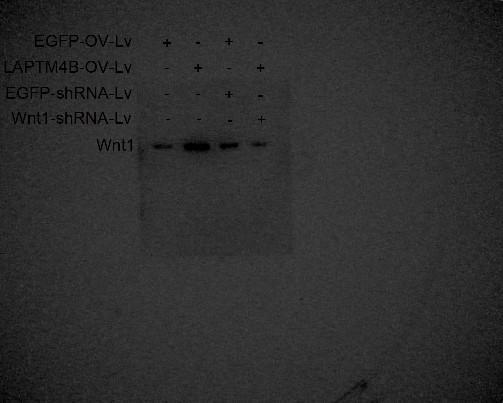

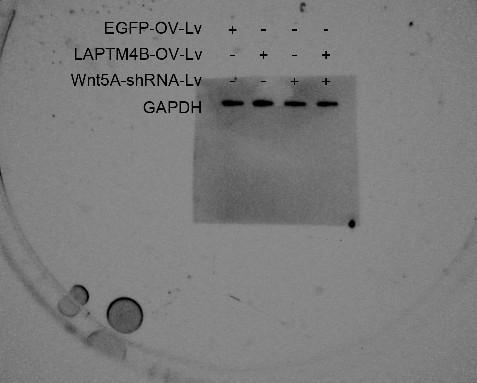

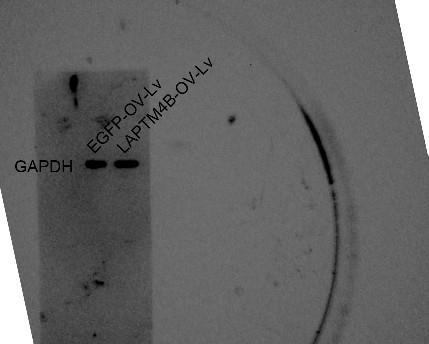

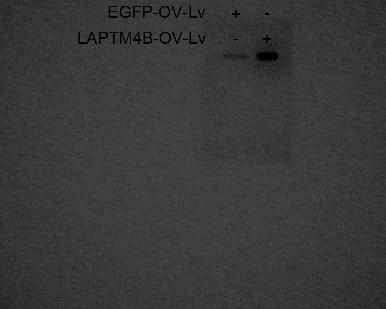

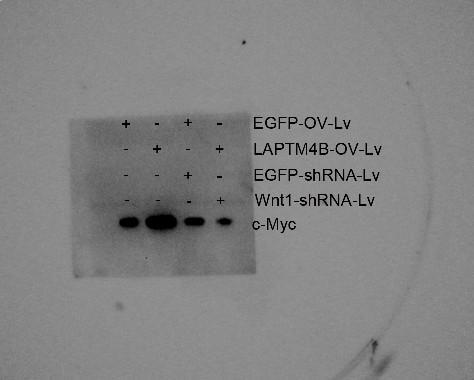

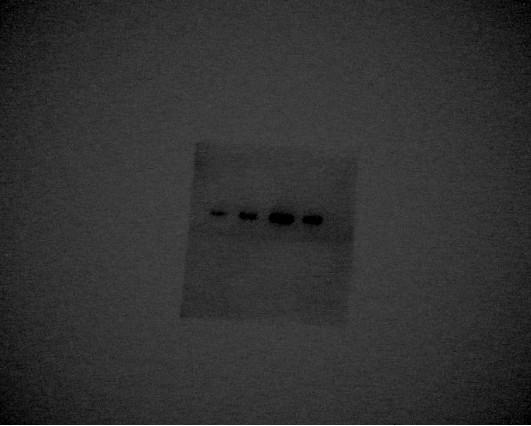

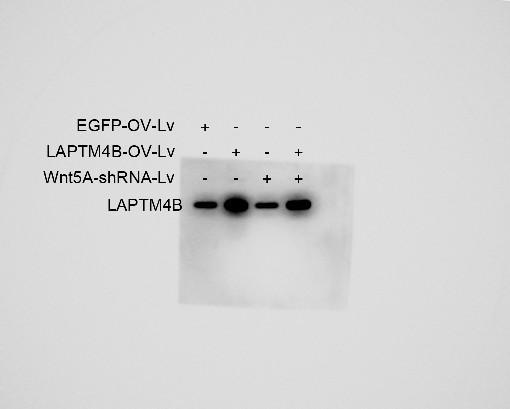

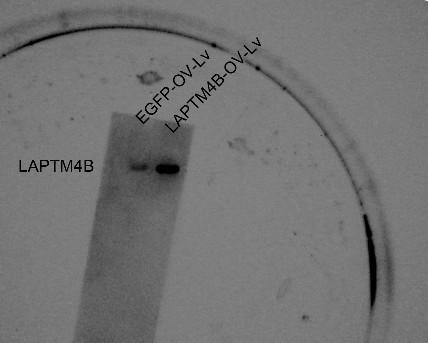


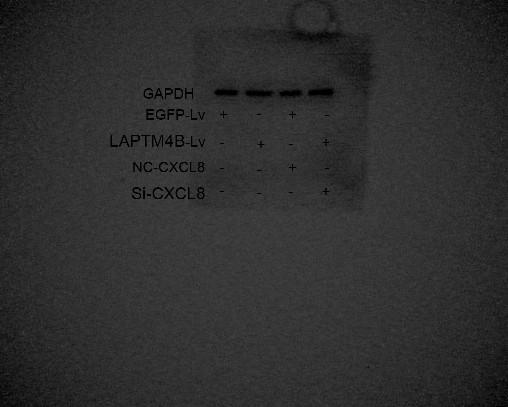

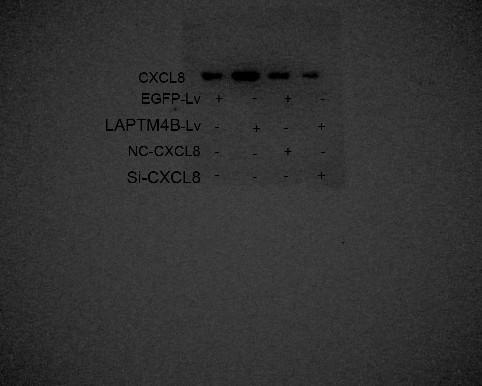

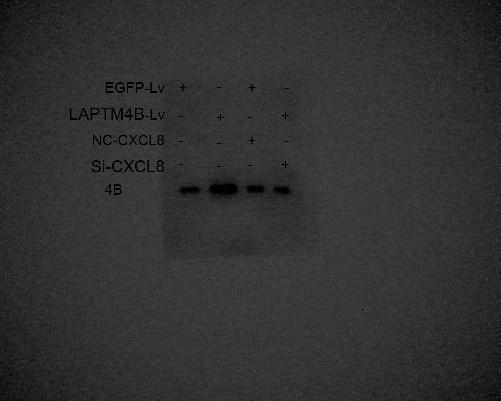


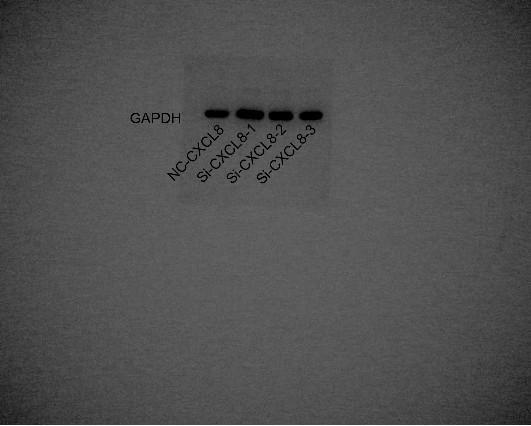

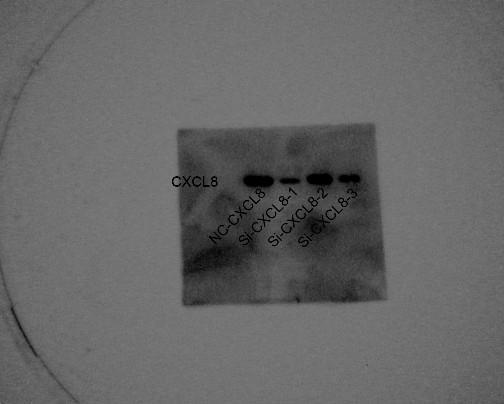

Supplement: Supplementary file 2 — Original Data [file 41419_2024_6542_MOESM2_ESM.docx]

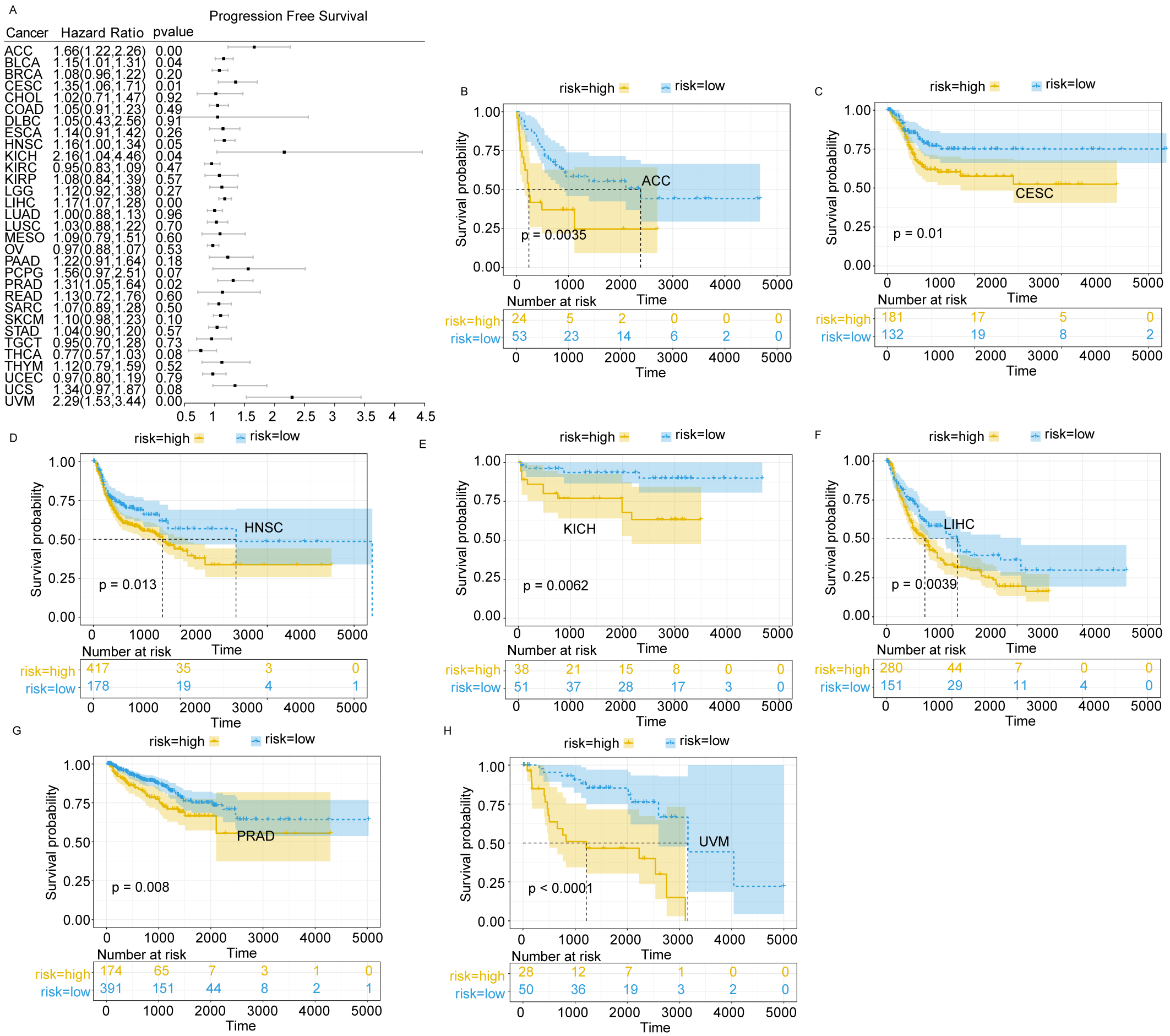

Supplement: Supplementary file 5 — Supplementary Figure1 [file 41419_2024_6542_MOESM5_ESM.tif]

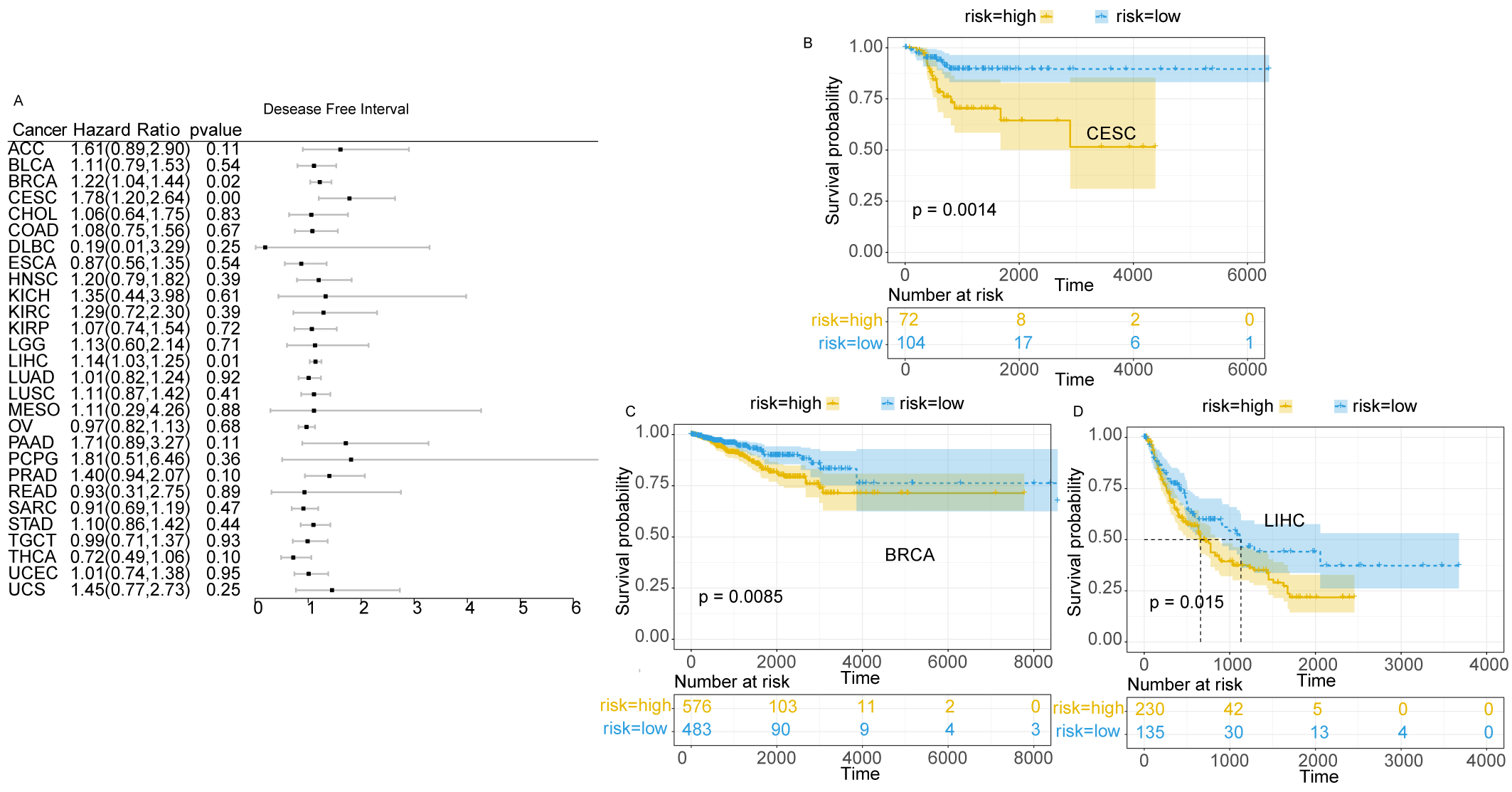

Supplement: Supplementary file 6 — Supplementary Figure2 [file 41419_2024_6542_MOESM6_ESM.tif]

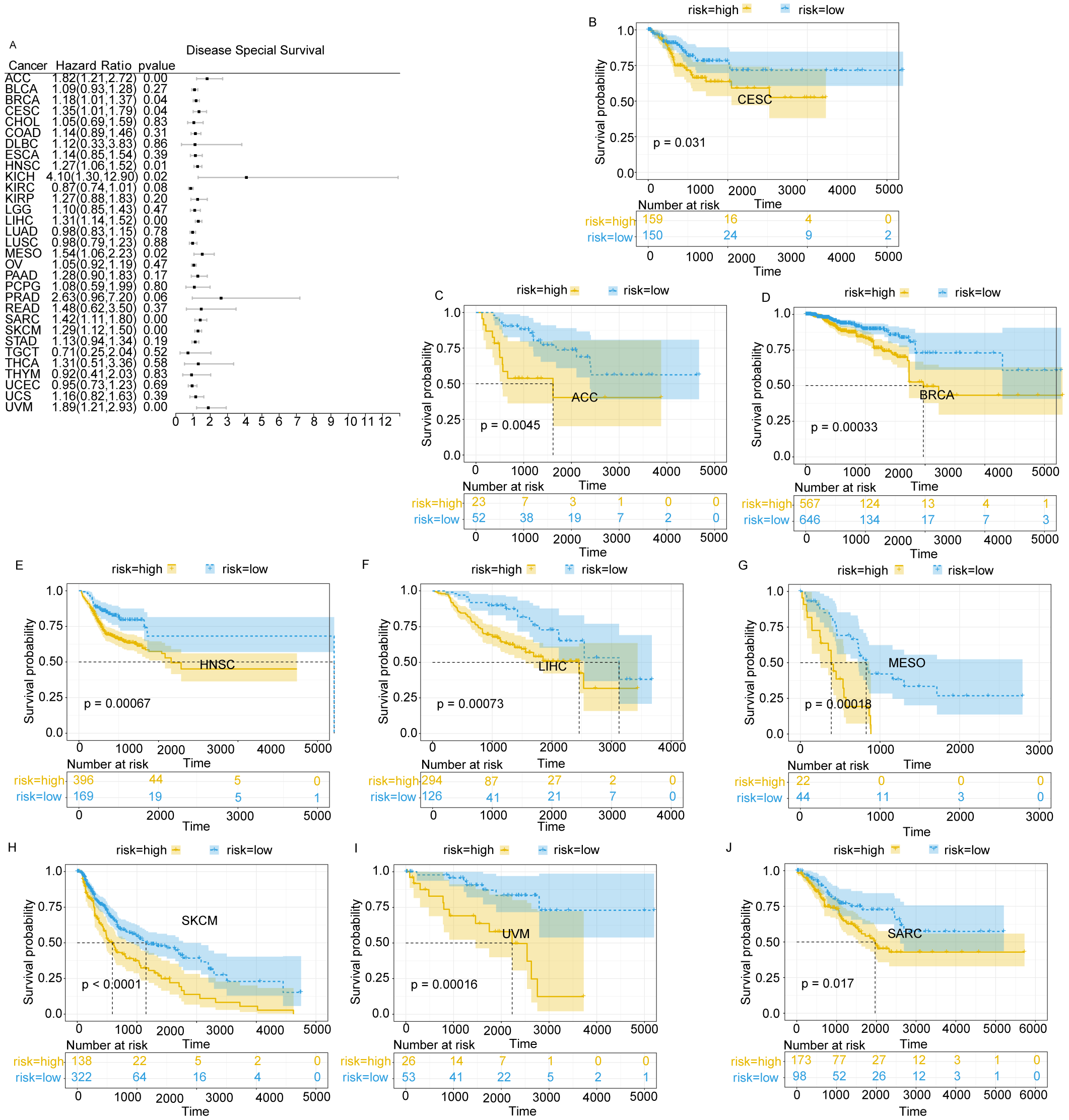

Supplement: Supplementary file 7 — Supplementary Figure3 [file 41419_2024_6542_MOESM7_ESM.tif]

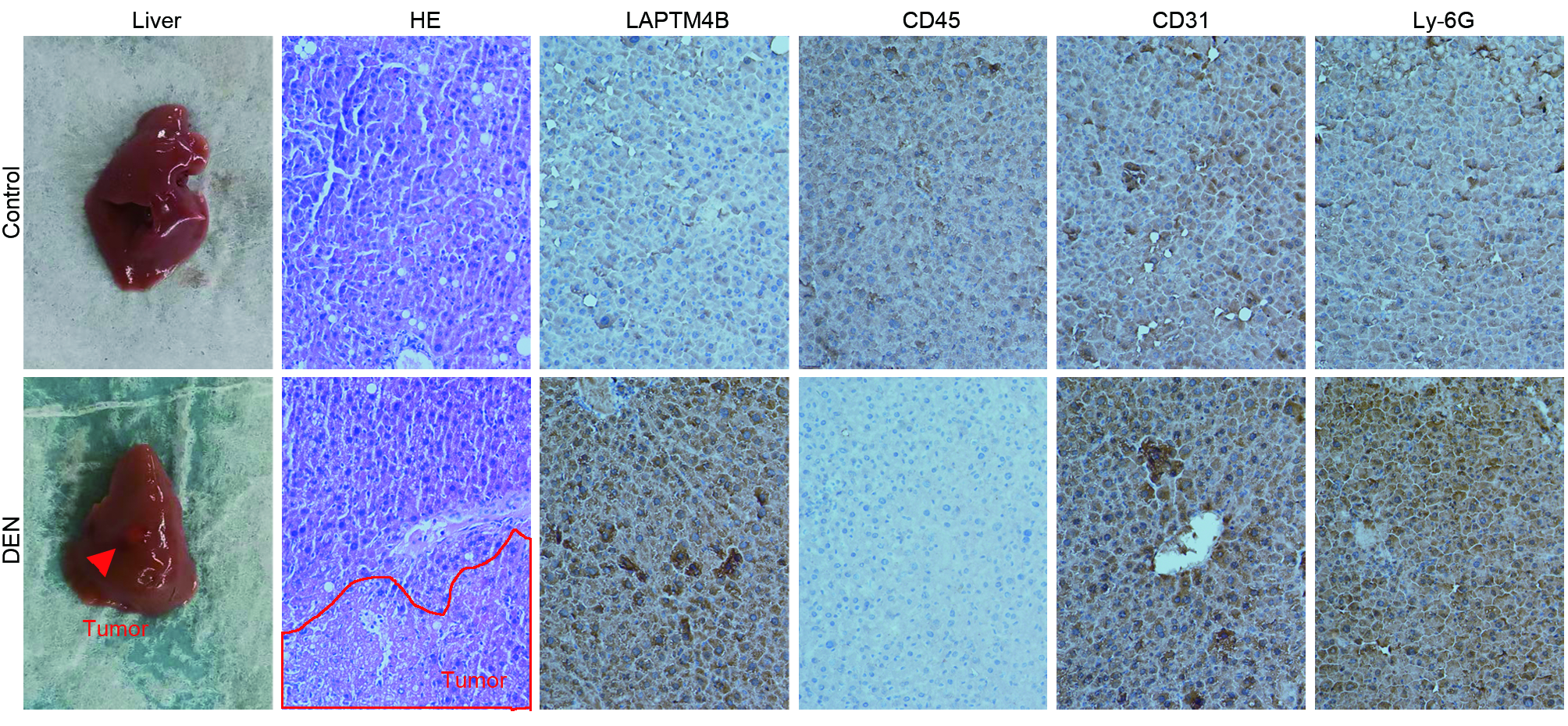

Supplement: Supplementary file 8 — Supplementary Figure4 [file 41419_2024_6542_MOESM8_ESM.tif]

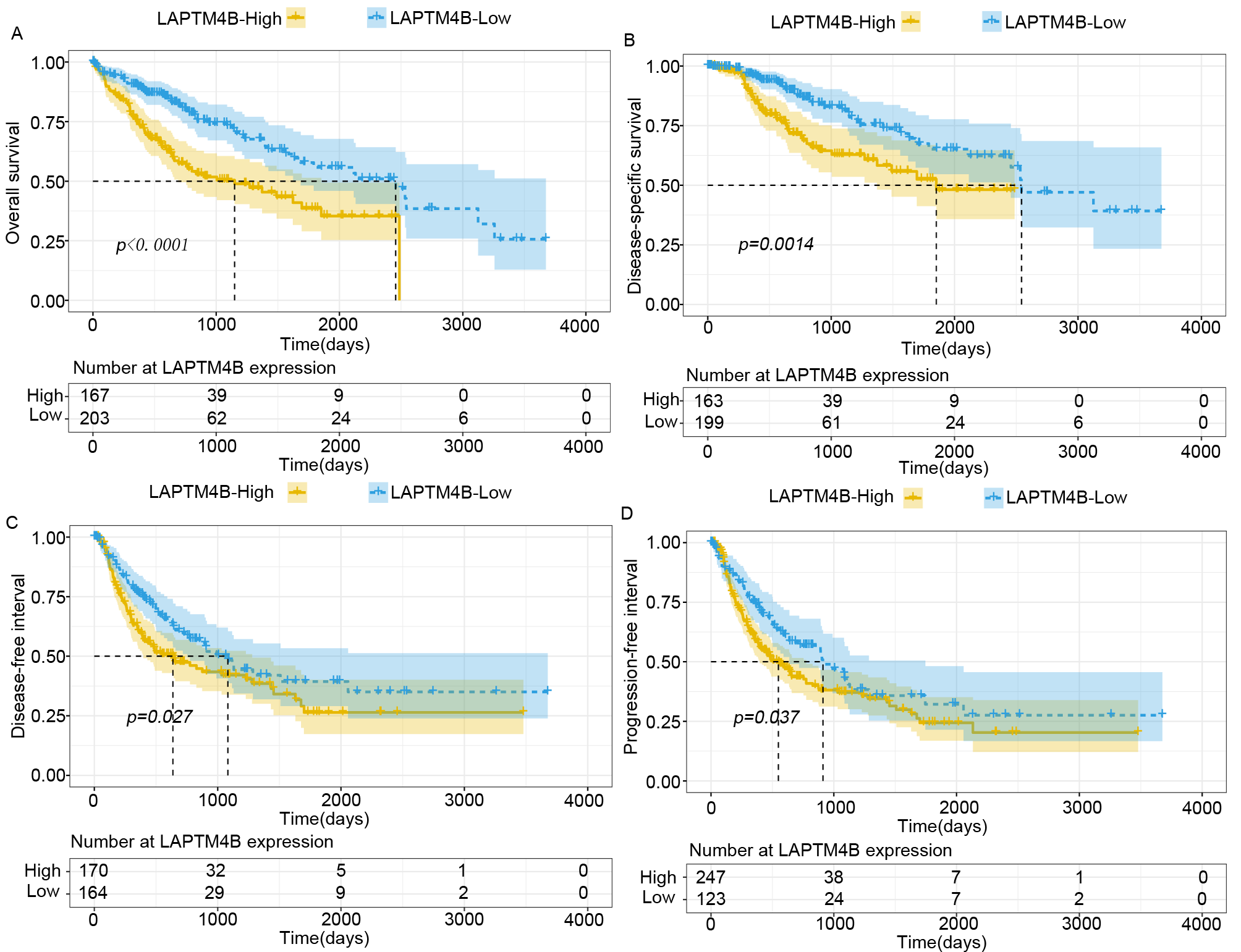

Supplement: Supplementary file 9 — Supplementary Figure5 [file 41419_2024_6542_MOESM9_ESM.tif]

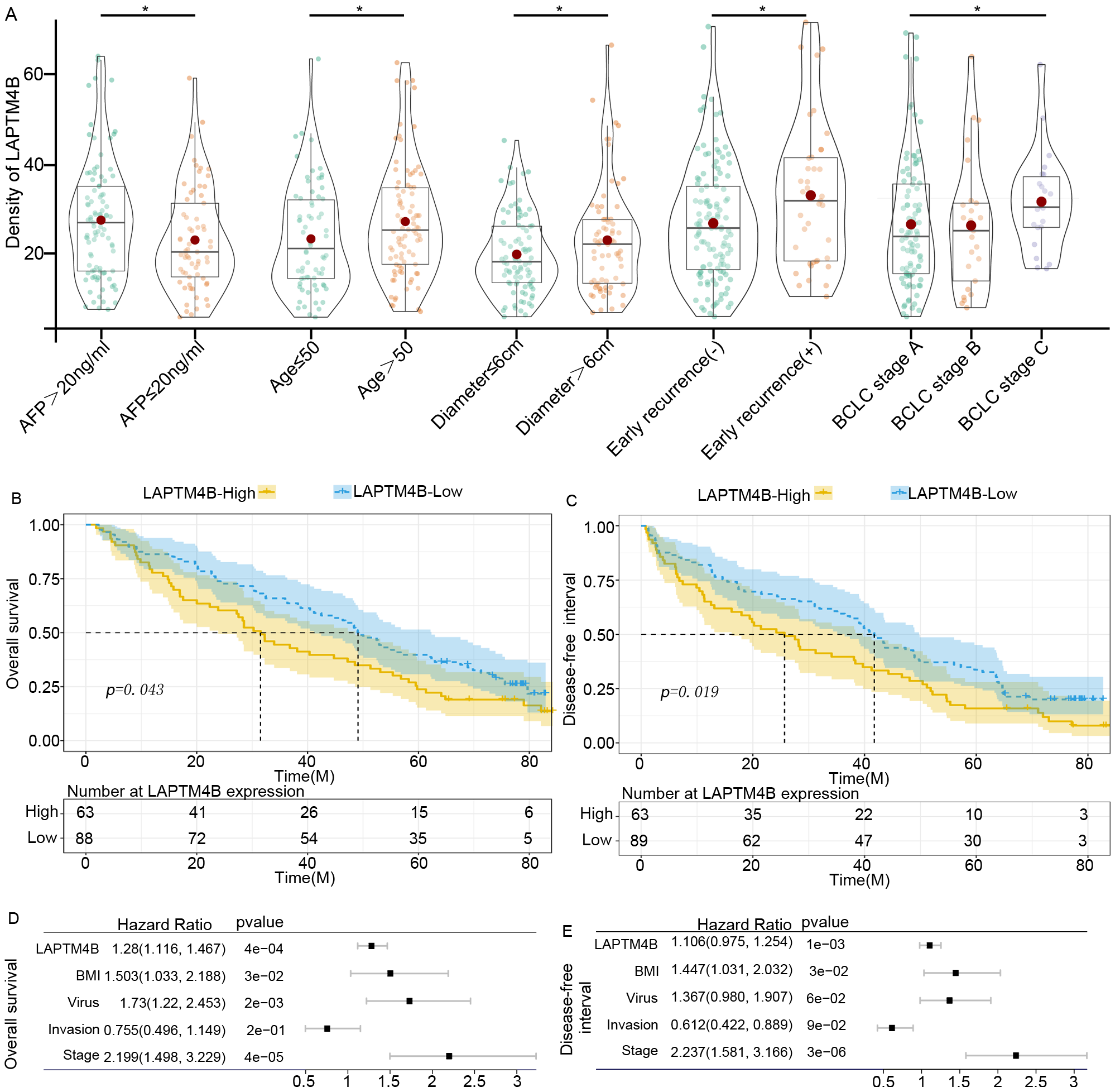

Supplement: Supplementary file 10 — Supplementary Figure6 [file 41419_2024_6542_MOESM10_ESM.tif]

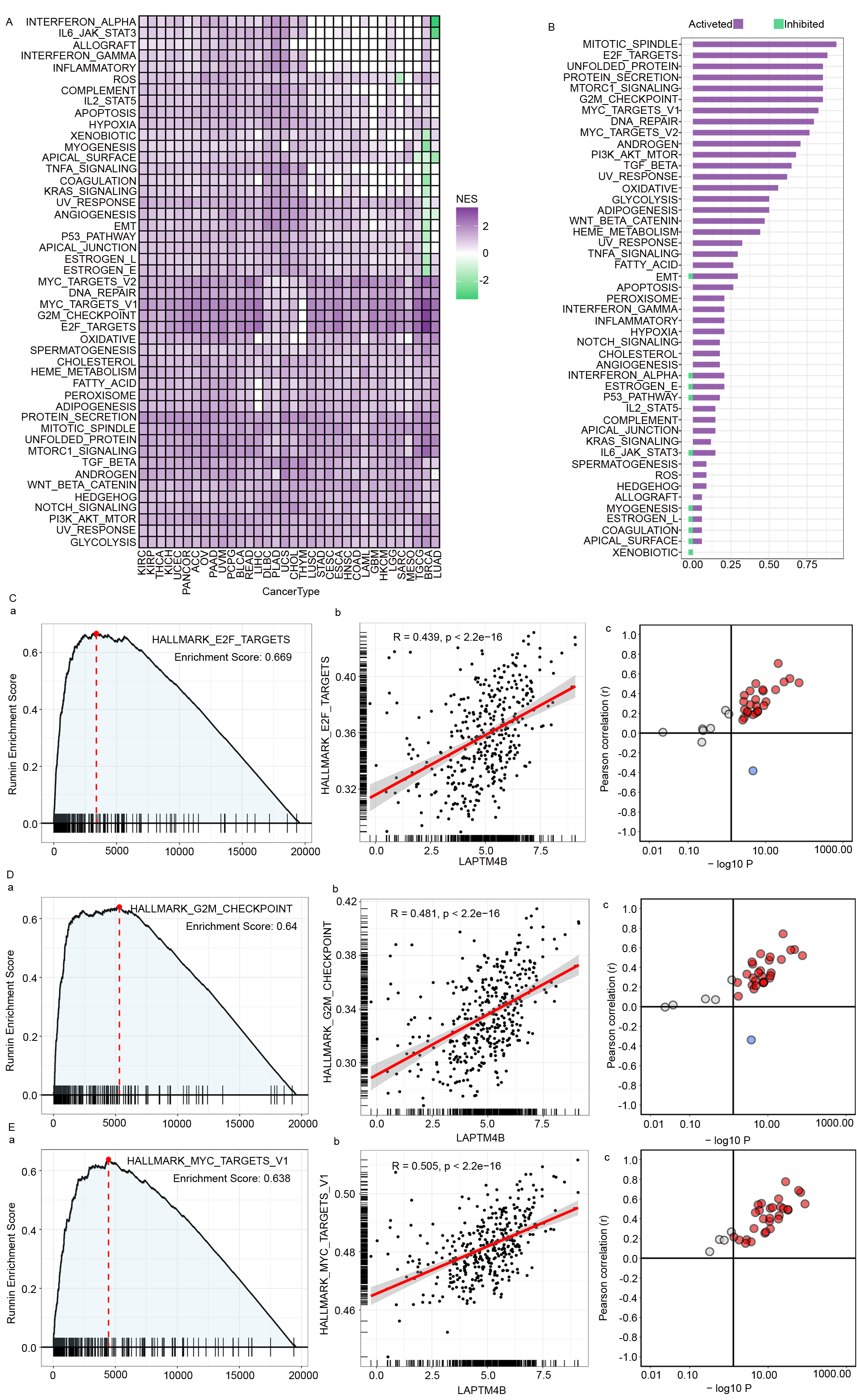

Supplement: Supplementary file 11 — Supplementary Figure7 [file 41419_2024_6542_MOESM11_ESM.tif]
