# Supplementary material for: LAPTM4B-mediated hepatocellular carcinoma stem cell proliferation and MDSC migration: implications for HCC progression and sensitivity to PD-L1 monoclonal antibody therapy
Source: Cell Death Dis. 2024 Feb 22;15(2):165. doi: 10.1038/s41419-024-06542-8 (PMC10884007; doi:10.1038/s41419-024-06542-8)
Supplement: Supplementary file 3 — Antibody colonies used in this article [file 41419_2024_6542_MOESM3_ESM.docx]

| Supplementary Table 1. Antibody colonies used in this article | | |
| --- | --- | --- |
| Antibody colonies | Source | Identifier |
| LAPTM4B Polyclonal antibody | Proteintech | 18895-1-AP |
| Anti-ER81/ETV1 antibody | Abcam | ab314874 |
| c-MYC Monoclonal antibody | Proteintech | 67447-1-Ig |
| Beta Catenin Monoclonal antibody | Proteintech | 66379-1-Ig |
| WNT3A Rabbit pAb | ABclonal | A0642 |
| WNT1 Rabbit pAb | ABclonal | A2475 |
| FITC-conjugated Goat Anti-Rabbit IgG | Elabscience | E-AB-1014 |
| FITC-conjugated Goat Anti-Mouse IgG | Elabscience | E-AB-1015 |
| TRITC-conjugated Goat Anti- Rabbit IgG | Elabscience | E-AB-1053 |
| HRP-conjugated Goat Anti-Mouse IgG | Elabscience | E-AB-1008 |
| HRP-conjugated Goat Anti-Rabbit IgG | Elabscience | E-AB-1003 |
| Anti-Mouse Ly-6G (Gr-1) (RB6-8C5) | Proteintech | 65140-1-Ig |
| GAPDH Monoclonal Antibody | Proteintech | 60004-1-Ig |
| CoraLite® Plus 488 Anti-Mouse Ly-6G (1A8) | Proteintech | CL488-65078 |
| PE Anti-Mouse CD11b (M1/70) | Proteintech | PE-65055 |
| FITC Anti-Human CD4 (SK3) | Proteintech | FITC-65147 |
| PE Anti-Human CD8a (RPA-T8) | Proteintech | PE-65144 |
| CD45 Rabbit PolymAb® (A24802) | ABclonal | A24802 |
| Recombinant Anti-IL-8 antibody[EPR26511-74] | Abcam | ab289992 |
| Nuclear Extraction Kit | Abcam | ab113474 |
| CD31 Polyclonal antibody | Proteintech | 11265-1-AP |
