# Supplementary material for: LAPTM4B-mediated hepatocellular carcinoma stem cell proliferation and MDSC migration: implications for HCC progression and sensitivity to PD-L1 monoclonal antibody therapy
Source: Cell Death Dis. 2024 Feb 22;15(2):165. doi: 10.1038/s41419-024-06542-8 (PMC10884007; doi:10.1038/s41419-024-06542-8)
Supplement: Supplementary file 12 — Supplementary Figure annotation [file 41419_2024_6542_MOESM12_ESM.docx]

Supplementary Figure 1 Impact of LAPTM4B expression on Progression-Free Survival (PFS) in different cancer patients

A: Forest plot of overall survival in different tumor types.

B-H: Impact of high LAPTM4B expression on PFS in different tumors patients.

Supplementary Figure 2 Impact of LAPTM4B expression on Disease Free Interval (DFI) in different cancer patients

A: Forest plot of DFI in different tumor types.

D: Impact of high LAPTM4B expression on DFI in different tumors patients.

Supplementary Figure 3 Impact of LAPTM4B expression on Disease Specific Survival (DSS) in different cancer patients

A: Forest plot of DSS in different tumor types.

J: Impact of high LAPTM4B expression on DSS in different tumors patients.

Supplementary Figure 4 Mouse hepatocellular carcinoma model

The model construction method can be found in the Methods section.Tissue specimens were subjected to Hematoxylin and Eosin (HE) staining and immunohistochemistry, followed by examination and imaging under a microscope.

Supplementary Figure 5 Survival curves for patients with high and low LAPTM4B expression

A: The impact of LAPTM4B expression on overall survival (OS) of hepatocellular carcinoma (HCC) patients in the TCGA database.

B: The impact of LAPTM4B expression on Disease-Specific Survival (DSS) of hepatocellular carcinoma (HCC) patients in the TCGA database.

C: The impact of LAPTM4B expression on Disease-Free Interval (DFI) of hepatocellular carcinoma (HCC) patients in the TCGA database.

D: The impact of LAPTM4B expression on Progression-Free Interval（PFI） of hepatocellular carcinoma (HCC) patients in the TCGA database.

Supplementary Figure 6 Clinical information from patients at the Eastern Hepatobiliary Hospital

A: Violin plot showing the correlation between LAPTM4B density and clinical indicators in patients.

B: Overall survival (OS) curves for patients with high and low LAPTM4B expression.

C: Disease-Free Interval (DFI) curves for patients with high and low LAPTM4B expression.

D: Risk factors affecting patient Overall survival (OS).

E: Risk factors affecting patient Disease-Free Interval (DFI).

Supplementary Figure 7 Metabolic pathways involved in LAPTM4B

A: Heatmap illustrating the enrichment of metabolic pathways involving LAPTM4B in pan-cancer contexts.

B: Bar chart depicting enriched metabolic pathways involving LAPTM4B in hepatocellular carcinoma (HCC).

C: Enrichment analysis of LAPTM4B with E2F_TARGETS.

D: Enrichment analysis of LAPTM4B with G2M_CHECKPOINT.

E: Enrichment analysis of LAPTM4B with MYC_TARGETS.
